# The metal-binding GTPases CobW2 and CobW3 are at the crossroads of zinc and cobalt homeostasis in *Cupriavidus metallidurans*

**DOI:** 10.1128/jb.00226-24

**Published:** 2024-07-23

**Authors:** Diana Galea, Martin Herzberg, Dietrich H. Nies

**Affiliations:** 1 Molecular Microbiology, Institute for Biology/Microbiology, Martin-Luther-University Halle-Wittenberg, Halle (Saale), Germany; 2 Department of Environmental Analytical Chemistry, Helmholtz Centre for Environmental Research – UFZ, Leipzig, Germany; Queen Mary University of London, London, United Kingdom

**Keywords:** zinc, cobalt, *Cupriavidus metallidurans*

## Abstract

The metal-resistant beta-proteobacterium *Cupriavidus metallidurans* is also able to survive conditions of metal starvation. We show that zinc-starved cells can substitute some of the required zinc with cobalt but not with nickel ions. The zinc importer ZupT was necessary for this process but was not essential for either zinc or cobalt import. The cellular cobalt content was also influenced by the two COG0523-family proteins, CobW2 and CobW3. Pulse-chase experiments with radioactive and isotope-enriched zinc demonstrated that both proteins interacted with ZupT to control the cellular flow-equilibrium of zinc, a central process of zinc homeostasis. Moreover, an antagonistic interplay of CobW2 and CobW3 in the presence of added cobalt caused a growth defect in mutant cells devoid of the cobalt efflux system DmeF. Full cobalt resistance also required a synergistic interaction of ZupT and DmeF. Thus, the two transporters along with CobW2 and CobW3 interact to control cobalt homeostasis in a process that depends on zinc availability. Because ZupT, CobW2, and CobW3 also direct zinc homeostasis, this process links the control of cobalt and zinc homeostasis, which subsequently protects *C. metallidurans* against cadmium stress and general metal starvation.

**IMPORTANCE:**

In bacterial cells, zinc ions need to be allocated to zinc-dependent proteins without disturbance of this process by other transition metal cations. Under zinc-starvation conditions, *C. metallidurans* floods the cell with cobalt ions, which protect the cell against cadmium toxicity, help withstand metal starvation, and provide cobalt to metal-promiscuous paralogs of essential zinc-dependent proteins. The number of cobalt ions needs to be carefully controlled to avoid a toxic cobalt overload. This is accomplished by an interplay of the zinc importer ZupT with the COG0523-family proteins, CobW3, and CobW2. At high external cobalt concentrations, this trio of proteins additionally interacts with the cobalt efflux system, DmeF, so that these four proteins form an inextricable link between zinc and cobalt homeostasis.

## INTRODUCTION

*Cupriavidus metallidurans* is a beta-proteobacterium that is adapted to metal-rich environments such as zinc deserts and auriferous soils (1–4). *C. metallidurans* strain CH34 contains a chromosome, a chromid, and two large plasmids with a variety of metal-resistance determinants (5, 6). The gene products of these determinants export excessively high concentrations of transition metal ions from the cytoplasm to the periplasm, and others from there to the outside. In the case of some metals, reduction and oxidation reactions to less toxic species are also mechanisms employed to decrease the toxic burden of the respective metal ion or complex (7).

Cobalt and zinc ions can be imported into the *C. metallidurans* cell by a variety of import systems (8–10), for instance, secondary, *proton motive force*-driven members ZupT of the ZIP [ZRT/IRT, TC2.A.5 (11, 12)] and CorA1, CorA2, and CorA3 of the MIT (metal inorganic transport, TC1.A.35) families. The name of these latter transport systems stems from the fact that a deletion of the cognate gene results in increased cobalt tolerance (13, 14). When the cytoplasmic content of zinc or cobalt ions becomes too high, Zn(II) is exported by the P_IB2_-type ATPases ZntA and CadA, and Co(II) is exported by the CDF (cation diffusion facilitator, TC2.A.4) protein, DmeF (Fig. S1) (15, 16). At even higher concentrations of these ions, the plasmid-encoded transenvelope efflux systems CzcCBA and CnrCBA export Zn(II) and Co(II) to the environment (7). Thus, export and import reactions adjust the cytoplasmic metal zinc concentration in a flow equilibrium, which is buffered by cytoplasmic metal-binding activities (17, 18). The expression “flow equilibrium” was used here to emphasize the importance of metal transport systems to reach the steady state condition of cellular zinc homeostasis.

Despite being a metal-resistant bacterium, *C. metallidurans* is also able to cope with metal-starvation conditions. In the case of zinc ion limitation, the Zur regulon mediates this ability (19–21). Products of this regulon are Zur itself, ZupT, and two metal-binding GTPases of the COG0523 family (20–24), CobW2 and CobW3 (Fig. S1). CobW2 is a zinc-storage compound that binds up to six Zn ions per polypeptide with low affinity at binding sites in the middle of the peptide chain and can unfold in the presence of MgGTP. CobW3 has no a GTPase activity but is able to sequester up to eight Zn ions per polypeptide with decreasing affinity to sites located at the carboxy terminus and equilibrates metal import by ZupT with that of other metal transport systems. When treated with a mixture of metal ions, CobW3 is also able to bind 2 Ni(II), 1 Co(II), and 1 Cd(II) instead of four Zn ions (20). Moreover, under even more extreme zinc starvation conditions, release of Zur from a double binding site at the *cobW1p* promoter allows expression of an operon encoding CobW1 as a third CobW protein, along with several paralogs of zinc-requiring enzymes and the metal-promiscuous GTP cyclohydrolase FolE_IB2 (20, 21, 25). FolE-type enzymes are important for the initiation of folate biosynthesis by cyclo-hydrolyzation of GTP, and synthesis of GTP needs tetrahydrofolate; hence, folate biosynthesis can be described as an “Achilles heel” of bacterial metabolism (26–28).

*C. metallidurans* possesses three FolE-type enzymes, the strictly zinc-dependent FolE_IA, and the metal-promiscuous FolE_IB1 and FolE_IB2 proteins. FolE_IB1 and IB2 are needed for growth under zinc-starvation conditions (25). The optimally suited cofactors for the two FolE_IBs are Fe(II), Mn(II), and Co(II) (25); however, *C. metallidurans* contains only a very low number of Mn atoms per cell, does not have a NRAMP-type manganese importer (TC2.A.55), and lacks a Mn-dependent superoxide dismutase (8, 29). Iron, on the other hand, is used for a multitude of biochemical reactions (30), but uncontrolled Fe(II) can be extremely detrimental due to its ability to catalyze the Fenton reaction (31–33).

This leaves Co(II) as the most suitable metal cofactor for the metal-promiscuous enzymes in *C. metallidurans* under zinc starvation conditions, besides cobalamin as an important cobalt sink (34). Nevertheless, too high intracellular cobalt concentrations are toxic and damage-nascent iron-sulfur clusters (35–37). If Co(II) can substitute for Zn(II), as discussed for *Salmonella* (38), it should be used only under zinc starvation conditions, and its cellular content must be strictly controlled. In addition to the overall affinity of a metal cation to a protein as defined by its rank in the Irving-Williams series (39), the actual availability controls metalation of a protein by a specific metal cation in competition with another metal cation (40, 41). Although CobW2 has been discussed to be a zinc-storing protein, CobW3 may be capable of sensing metal availability by binding the cations at its carboxy-terminal metal-binding site and use this information to control metal homeostasis by influencing activity of metal transport systems. It is therefore conceivable that the actual availability of cobalt and zinc could control metalation of CobW3 and, subsequently, the import of zinc and other metal cations.

This current study provides evidence for three processes that support the above proposal. First, *C. metallidurans* fills up a part of its cellular zinc pool with cobalt ions. ZupT and CobW3 control this response with a minor contribution from CobW2. Second, these three proteins are also involved in the cellular flow equilibrium of zinc by affecting zinc import rather than efflux. Third, disturbance of the zinc-cobalt homeostasis mediated by these three proteins results in decreased cobalt, cadmium, and metal-starvation tolerance, with DmeF supporting ZupT, CobW3, and CobW2 (Fig. S1) to mediate cobalt resistance.

## RESULTS

### Experimental strategy

Due to the fact that the GTP cyclohydrolase FolE_IB1 can be activated by Co(II) 50-times more effectively than by Ni(II) (25), *C. metallidurans* may import cobalt rather than nickel under zinc starvation conditions to metalate this enzyme and continue folate biosynthesis. To investigate whether zinc availability influences the cellular cobalt content, the cells were cultivated in Tris-buffered mineral salts medium TMM (5) with gluconate as the carbon source but with different zinc and cobalt concentrations (Table S1). In TMM, trace element solution SL6 (42) provided 35.3 nM Zn(II) and 84.1 nM Co(II) to the medium; however, the actual zinc content varies due to the zinc content of the respective NaSO_4_ source (25). This fact was used to design six TMM solutions with different zinc and cobalt concentrations exposing the *C. metallidurans* cells to different levels of zinc and cobalt starvation stress (Table S1). All strains used (Table S2) were derivatives of the plasmid-free strain AE104 because a ∆*zupT* deletion results in curing of the plasmid pMOL30 in *C. metallidurans* CH34 wild type (43). All experiments were performed at least as three biological repeats. For most experiments, data points were judged as different if their ratio was at least two and if their deviation bars did not touch or overlap.

### Zinc controls cobalt homeostasis in *C. metallidurans*

*C. metallidurans* strain AE104 was cultivated to the mid-exponential phase of growth (150 Klett units) in three TMM media with 200 nM (aZn), 64 nM (mZn), or 39 nM (lZn) Zn(II), respectively (Table 1). As determined by ICP-MS (inductively coupled plasma mass spectrometry), the zinc content of the cells decreased with the decreasing zinc content of the respective medium from 101,000 Zn per cell via 33,000 down to 7,100 when no SL6 was added to the medium. When more zinc (1, 10, and 100 µM) was added to these cells before the mid-exponential phase of growth, their zinc content increased. At high zinc concentrations, the zinc ion content of the cells that had been cultivated under these conditions was similar and reached an intracellular saturation of the zinc repository with on average 150,000 Zn ions per cell, which is comparable with previous studies (17).

**TABLE 1:** Metal content of *C. metallidurans* strain AE104 in different TMM media^a^

Addition	Atoms per cell						
	Mg, 10^6^	Ca, 10^3^	Fe, 10^3^	Zn, 10^3^	Co, 10^3^	Ni, 10^3^	Cu, 10^3^
TMM 200 nM Zn (aZn)							
0 µM Zn	13.4 ± 0.7	251 ± 9	998 ± 4	101 ± 4	5.3 ± 0.4	4.2 ± 0.2	5.6 ± 0.4
1 µM Zn	14.8 ± 0.6	231 ± 27	1,082 ± 14	128 ± 8	4.7 ± 0.3	4.4 ± 0.2	4.7 ± 0.2
10 µM Zn	14.1 ± 0.3	197 ± 18	1,059 ± 33	124 ± 4	4.4 ± 0.2	4.0 ± 0.0	4.7 ± 0.3
100 µM Zn	17.6 ± 0.4	139 ± 8	834 ± 37	157 ± 19	4.4 ± 0.0	3.4 ± 0.0	5.1 ± 0.2
TMM 64 nM Zn(II) (mZn)							
0 µM Zn	11.2 ± 0.3	269 ± 166	819 ± 28	33.4 ± 5.4	20.5 ± 0.9	5.0 ± 0.6	4.3 ± 0.7
1 µM Zn	11.9 ± 0.2	280 ± 131	874 ± 10	115 ± 1	18.4 ± 1.6	3.6 ± 0.4	4.2 ± 0.4
10 µM Zn	12.6 ± 0.4	209 ± 74	863 ± 33	160 ± 4	21.4 ± 1.3	3.9 ± 0.2	4.1 ± 0.3
100 µM Zn	14.3 ± 0.5	120 ± 66	641 ± 19	155 ± 10	17.7 ± 0.7	2.8 ± 0.3	4.3 ± 1.1
TMM 39 nM Zn(II), no SL6 (lZn)							
0 µM Zn	13.3 ± 0.4	193 ± 43	1,003 ± 40	7.1 ± 1.2	0.3 ± 0.0	4.2 ± 0.1	2.8 ± 0.6
1 µM Zn	13.0 ± 0.5	277 ± 98	850 ± 52	113 ± 8	0.2 ± 0.0	3.3 ± 0.2	5.3 ± 3.4
10 µM Zn	13.9 ± 0.6	187 ± 48	840 ± 45	138 ± 9	0.2 ± 0.0	3.1 ± 0.2	2.4 ± 0.2
100 µM Zn	16.6 ± 0.7	142 ± 43	709 ± 14	186 ± 18	0.2 ± 0.0	2.6 ± 0.1	2.8 ± 0.5

^*a*^ *C. metallidurans* AE104 was cultivated in TMM medium with ambient zinc (aZn) TMM, which leads to fully zinc-replete cells, under moderate zinc starvation (mZn) and under low zinc and cobalt (lZn) to a turbidity of 100 Klett, before mid-exponential phase of growth. Zn(II) was added at the indicated concentration. Incubation was continued with shaking to a turbidity of 150 Klett units reaching the mid-exponential phase and the metal content was determined by ICP-MS. *n* = 3, with the standard deviations indicated.

Although no important changes between the intracellular content of Mg, Ca, Fe, Ni, and Cu could be observed (Table 1), the cobalt content depended on the medium zinc or cobalt concentration. Cells grown in the presence of the same cobalt concentration (86 nM) with 200 nM (aZn) zinc contained ~5,000 Co ions per cell, but with 64 nM Zn (mZn) about 20,000, and those in low zinc and low cobalt (~2 nM) medium only a few hundred Co atoms per cell were detected. Addition of zinc (1, 10, or 100 µM) before reaching the mid-exponential phase of growth did not change the cobalt content of the cells in this incubation experiment in indirect proportion to the zinc content. The cells seemed to have accumulated more cobalt under zinc starvation condition (mZn) than under zinc-replete conditions (aZn), provided sufficient cobalt (86 nM) was available in these growth media, which was not the case in media without SL6 (lZn) (Table 1). The threshold for the zinc concentration that stimulated increased cobalt accumulation should be between 64 nM and 200 nM Zn(II) but was far below 1 µM Zn(II). Zinc starvation controlled the cobalt level of the cells, but the starvation stress had to be present at the beginning of growth, or even in the pre-cultures. It took some time for the cells to experience starvation conditions and react to them.

To obtain a better measure for the threshold concentration of zinc that governs the cobalt level, strain AE104 was cultivated in standard TMM (160 nM Zn), high-zinc (400 nM Zn), and low zinc-cobalt medium (39 nM Zn) without trace element solution SL6 (Table 2). As published previously (8, 17, 21, 43, 44), the zinc content of the parental strain AE104 remained at about 70,000 to 80,000 Zn ions per cell in standard TMM, which included 160 nM Zn(II) (Table 2). The increased zinc concentration in high zinc medium with 400 nM Zn(II) did not change this number in AE104, although the deviation of this number decreased by half compared with the standard medium.

**TABLE 2:** Zinc and cobalt content of *C. metallidurans* strain AE104 and its ∆*zupT* mutant under various growth conditions^*a*^

Strain	Medium		1,000 Metal atoms per cell	
	Zn (nM)	Co (nM)	Co	Zn
High-zinc TMM (hZn)				
AE104	400	110	3.7 ± 1.1	75.8 ± 7.7
∆*zupT*	400	110	6.4 ± 0.9	42.8 ± 4.3
Standard TMM				
AE104	160	110	21.3 ± 10.7	68.4 ± 12.4
∆*zupT*	160	110	7.3 ± 1.9	28.6 ± 4.8
Low-zinc-cobalt, no SL6 (lZn)				
AE104	39	2	0.153 ± 0.045	27.5 ± 11.0
AE104	39	100	40.2 ± 3.6	18.1 ± 9.6
AE104	39	150	40.0 ± 7.4	19.7 ± 5.8
AE104	39	300	57.2 ± 16.5	21.6 ± 8.7
AE104	190	100	25.6 ± 3.6	83.6 ± 6.1
AE104	190	150	20.6 ± 3.5	65.4 ± 4.7
AE104	190	300	24.9 ± 3.2	74.5 ± 8.2
∆*zupT*	39	2	0.105 ± 0.073	19.9 ± 2.4
∆*zupT*	39	100	35.2 ± 3.3	14.6 ± 2.2
∆*zupT*	39	150	44.8 ± 5.9	14.5 ± 6.2
∆*zupT*	39	300	46.5 ± 19.8	37.1 ± 8.5
∆*zupT*	190	100	5.4 ± 0.5	32.5 ± 2.8
∆*zupT*	190	150	8.2 ± 0.8	23.3 ± 3.5
∆*zupT*	190	300	14.1 ± 4.6	25.8 ± 4.4

^*a*^ Same cultivation conditions as in Table 1. The metal content was verified by ICP-MS and was in the expected range. Data for all metals are presented in Table S3

Again, strain AE104 accumulated approximately 20,000 Zn ions per cell in low Zn-Co medium (lZn) (Tables 1 and 2). This was not surprising because 39 ± 8 nM zinc in low zinc/cobalt medium was distributed among 1.2 × 10^12^ cells L^−1^ (around the mid-exponential phase of growth), resulting in a maximal number of 19,500 ± 4,000 Zn ions per cell. In low-zinc medium, the zinc content may have been exhausted after growth. A similar calculation for the zinc content of moderate zinc medium of 160 ± 36 nM distributed among the cells calculates to 80,000 ± 18,000 Zn ions per cell. Again, the zinc content of the zinc growth medium was mirrored by that of the cellular zinc content. In contrast, the cellular zinc content remained at 76,000 Zn ions per cell in high zinc medium (400 nM, Table 2). The cells limited their cellular zinc content, e.g., by ZntA-mediated zinc efflux. They were under zinc starvation in low Zn-Co medium, exactly at the edge of zinc starvation at 160 nM zinc but were supplied with sufficient zinc at 400 nM zinc in the medium (Table 2).

A complete distribution of the 2 nM cobalt ions in low zinc/cobalt medium would result in about 1,000 Co ions per cell. The cobalt content of these cells was even below this number (Table 2). In moderate and high zinc media, complete distribution of the approximately 100 nM Co would give 50,000 Co ions per cell. The measured level was below this number, about 21,000 Co ions per cell in moderate zinc and only about 3,700 Co ions per cell in high zinc medium. When on the verge of zinc starvation, the cells accumulated a higher number of Co ions; however, accumulation did not exhaust the cobalt content of the growth medium. Zinc availability in the growth medium did indeed prove to control the cellular cobalt content.

**Fig 1:**
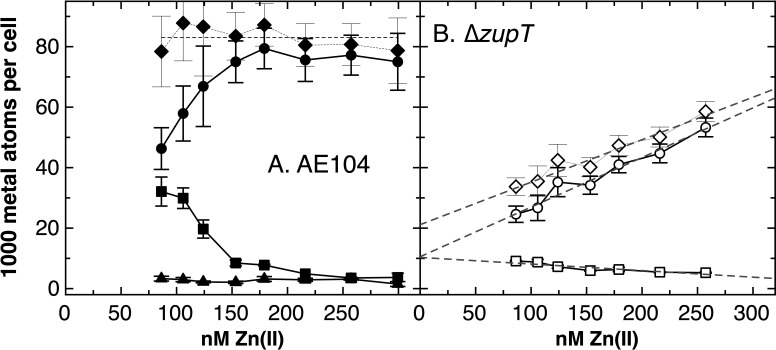
Zinc availability controls the cellular cobalt content in *C. metallidurans* AE104 and ZupT is required for this process. Cells were cultivated in moderate zinc mZn-TMM with an adjusted zinc content to the exponential phase of growth. The cellular zinc (circles), cobalt (squares), and nickel (triangles) contents were measured by ICP-MS. The diamonds represent the sum of the zinc plus cobalt contents per cell. Panel A. The *C. metallidurans* parental strain, AE104. The dashed line indicates the mean value of the sum of the zinc and cobalt content of 83,000 ± 3,900 (Zn + Co)/cell. Panel B. The cellular zinc content of the ∆*zupT* strain (open symbols) was fitted to the zinc availability with 11,300 ± 2,500 Zn/cell + 161 ± 15 zn/cell * nM medium zinc (98%), the cobalt content was 10,600 ± 800 Co/cell – 23 ± 5 Co/cell * nM medium zinc (91%),, and the Zn + Co content was 21,100 ± 3,900 (Zn + Co)/cell + 140 ± 22 (Zn + Co)/cell * nM medium zinc (97%). These functions are shown with dashed gray lines.

To test this observation further, strain AE104 was cultivated in TMM adjusted to concentrations between 85 nM and 300 nM Zn(II), and the metal content of medium and cells was determined by ICP-MS (Fig. 1A). The cellular zinc content increased with increasing metal availability and followed a curve that reached saturation at 150 nM Zn(II) in the growth medium. The cellular cobalt content, in contrast, followed an inverse curve. The sum of both metal contents was 83,000 ± 3,900 Zn + Co ions per cell. The cells filled up their cobalt content to a level of 83,000 ions per cell minus the zinc content. Interestingly, the nickel content of the cells was not affected (Fig. 1A, triangles). This demonstrated clearly that the zinc availability in the growth medium controlled the cobalt, but not the nickel, content in *C. metallidurans* strain AE104. This may allow cobalt ions to substitute for zinc ions and reflects a possible shared metal pool in the cell.

### The zinc importer ZupT of the ZIP protein family is involved in zinc-mediated control of the cellular cobalt content

When the ∆*zupT* mutant was incubated in TMM with adapted zinc concentrations (Fig. 1B), the zinc content of the mutant cells was lower than that of the parent; it did not follow a saturation curve but followed a linear fashion with the zinc content of the medium exhibiting an increase of 161 ± 17 Zn ions per cell per nM exogenously supplied Zn. The cobalt content decreased with the zinc content of the medium, corresponding to a decrease in 23 ± 5 Co ions per cell per nM Zn in the growth medium. Both lines crossed the y-axis at x = 0 with similar numbers, 11,300 ± 2,300 Zn and 10,600 ± 800 Co ions per cell. The total zinc plus cobalt content increased such that 140 ± 22 (Zn + Co) ions per cell per nM Zn represented the sum for the zinc and cobalt ion content (Fig. 1B).

The zinc content of the ∆*zupT* mutant cultivated in moderate zinc and low zinc/cobalt medium (Table 2) was at a value that could be expected from the linear function of the ion (Fig. 1B). In high zinc medium, the cells contained only 42,800 Zn ions per cell, much less than expected from the linear function. This indicated that efflux systems had been activated, for instance, ZntA. Nevertheless, ZupT was also required to adjust the cellular zinc content in high zinc medium. The cobalt content was also at the level expected based on the linear function (Fig. 1B) or from an exhaustive accumulation of the available cobalt in low zinc/cobalt medium (Table 2).

For a more detailed characterization of the role of ZupT in cobalt accumulation, the ∆*zupT* strain and its parent AE104 were cultivated in low zinc-cobalt (lZn) medium, which was supplemented with 100 nM, 150 nM, or 300 nM cobalt chloride, with or without additional 150 nM zinc chloride. This resulted in zinc concentrations in the growth medium of 39 nM or 190 nM (Table 2). At 39 nM Zn(II) in the medium, the cellular zinc content remained at the expected 20,000 Zn ions per cell with no difference between the ∆*zupT* strain and its parent; all available zinc had been accumulated. When Co(II) was added to the cells, both strains accumulated between 37,000 and about 50,000 Co ions per cell at 100 nM, 150 nM, or 300 nM Co(II) (Table 2). Again, the absence of ZupT made no difference with respect to the cellular cobalt content, despite the presence of the remaining import systems for divalent metal cations in the *C. metallidurans* cell. The cobalt content of the medium was not exhausted, although the Zn + Co content of 83,000 ions per cell had not been reached. Strain AE104 substituted Co for Zn only to between 50% and 70% of the total of 83,000 Zn + Co ions, however, not completely. ZupT was not needed under zinc starvation conditions at 39 nM Zn(II) for exhaustive zinc accumulation.

At 190 nM Zn(II), the zinc content of the AE104 parental strain was saturated between 70,000 and 80,000 Zn ions per cell (Fig. 1A; Table 2) but that of the ∆*zupT* mutant accumulated only between 23,000 and 32,000 Zn ions per cell (Table 2). This level was expected based on the linear dependence of the Zn content of ∆*zupT* cells on the zinc availability (Fig. 1B). In contrast to the parental strain, the Co content of ∆*zupT* cells remained very low when Co(II) was added to this medium. The Co content in ∆*zupT* increased in a linear fashion with the cobalt content of the medium but only in the presence of sufficient zinc (Table 2).

Under all these conditions, the cellular content of Ca, Mn, Fe, and Mo remained unchanged with some minor deviations of the copper content (Table S3). The nickel content was decreased at high cobalt concentrations. The magnesium content was increased by a factor of three in the ∆*zupT* mutant when grown in low zinc/cobalt medium that was supplemented with exogenous zinc and cobalt.

The largest difference between the zinc and cobalt content of the ∆*zupT* mutant and its parent was visible in standard TMM without any additions (Table 2). When Co(II) was added (Table 3), strain AE104 did not increase its cellular cobalt content when 1 µM Co(II) was added, but the level increased 2.7-fold when 5 µM Co(II) was added. The ∆*zupT* mutant cells started from a lower cobalt level of 7,000 Co ions per cell when cultivated without added Co and the level increased 3.4-fold and 8.7-fold when 1 µM or 5 µM Co(II) was added, respectively. Thereby, the ∆*zupT* cells attained a similar Co content to the parent cells. The zinc content of these cells decreased 0.42-fold in the ∆*zupT* mutant compared with AE104 when the cells were cultivated without added cobalt and remained at this level when cobalt was added (Table S4).

**TABLE 3:** Cobalt content of mutant strains cultivated in standard TMM*^a^*

Added Co(II), µM	0	1	5
Strains	1,000 Co per cell; Q (D)		
AE104	20.0 ± 8.7 (1.00; 0.0)	24.1 ± 2.0 (1.20; 0.4)	**54.7 ± 9.1 (2.73; 2.0)**
Δ*cobW3*	**3.9 ± 1.2 (0.19; 1.6)**	**14.9 ± 2.4 (3.84; 3.1)**	**46.3 ± 4.4 (11.9; 7.5)**
Δ*cobW2::dis*	13.3 ± 3.8 (0.66; 0.5)	**24.0 ± 6.4 (1.81; 1.1)**	**42.3 ± 3.2 (3.19; 4.2)**
Δ*cobW3* Δ*cobW2::dis*	**3.6 ± 0.7 (0.18; 1.8)**	**16.1 ± 2.4 (4.50; 4.0)**	**39.0 ± 3.5 (10.9; 8.4)**
			
Δ*zupT*	**7.3 ± 1.5 (0.36; 1.3)**	**24.9 ± 1.8 (3.42; 5.3)**	**63.4 ± 1.4 (8.70; 19)**
Δ*zupT* Δ*cobW3*	9.9 ± 1.0 (1.35; 1.0)	**30.0 ± 2.8 (3.04; 5.3)**	**62.7 ± 6.0 (6.36; 7.6)**
Δ*zupT* Δ*cobW2::dis*	7.2 ± 1.7 (0.99; 0.0)	**25.5 ± 0.7 (3.54; 7.6)**	**53.6 ± 3.2 (7.43; 9.4)**
Δ*zupT* Δ*cobW3* Δ*cobW2::dis*	7.9 ± 0.7 (1.09; 0.3)	**24.7 ± 2.5 (3.11; 5.2)**	**60.9 ± 4.0 (7.67; 11)**
			
Δ*dmeF*	20.8 ± 6.6 (1.04; 0.0)	**72.1 ± 7.0 (3.47; 3.8)**	**129.7 ± 13.7 (6.24; 5.4)**
Δ*dmeF* Δ*cobW3*	**5.1 ± 0.7 (0.24; 2.2)**	**42.1 ± 1.1 (8.29; 21)**	**119.0 ± 10.7 (23.4; 10)**
Δ*dmeF* Δ*cobW2::dis*	14.8 ± 4.2 (0.71; 0.6)	**62.6 ± 2.8 (4.24; 6.8)**	**149.9 ± 12.7 (10.2; 8.0)**
Δ*dmeF* Δ*cobW3* Δ*cobW2::dis*	**6.0 ± 1.1 (0.29; 1.9)**	**45.3 ± 1.8 (7.58; 14)**	**140.7 ± 6.7 (23.5; 17)**
			
Δ*dmeF* Δ*zupT*	16.4 ± 1.9 (0.82; 0.3)	**55.8 ± 5.0 (3.40; 5.7)**	n.d.
Δ*dmeF* Δ*zupT* Δ*cobW3*	18.1 ± 1.2 (1.10; 0.6)	**55.5 ± 3.3 (3.07; 8.5)**	n.d.
Δ*dmeF* Δ*zupT* Δ*cobW2::dis*	13.4 ± 1.5 (0.82; 0.9)	**55.1 ± 8.6 (4.10; 4.1)**	n.d.
Δ*dmeF* Δ*zupT* Δ*cobW3* Δ*cobW2::dis*	12.8 ± 2.4 (0.78; 0.8)	**81.7 ± 7.9 (6.36; 6.7)**	n.d.

^*a*^ The cells were cultivated in standard TMM medium (160 nM Zn) with and without added Co(II). The metal content was measured using the ICP-MS, and the cobalt content of the cells in 1,000 Co per cell is shown. The experiment was repeated with three biological replicates, and standard deviations are indicated. The value is followed by a ratio D of the cobalt contents of the cells followed by a D value in parentheses. In cells cultivated without added cobalt, the Co contents of the ∆*zupT* and the ∆*dmeF* mutants were compared with the value for the parental strain, AE104. The content for the ∆*cobW* deletion mutants was compared with the value of the respective AE104, ∆*zupT,* or ∆*dmeF* strains. For cells cultivated with added Co, the values were compared with that of the respective mutant strain grown without added Co. Given is the ratio Q and the D-value in parentheses. The full metal table is provided in the Supplement. Bold faced values indicate (0.66 < Q OR Q > 1.5) and (D > 1). Three biological repeats were performed; n.d., not done because of the high sensitivity of the respective strains.

### CobW2 and CobW3 control cobalt homeostasis in *C. metallidurans*

In cells cultivated in standard TMM, the absence of the gene encoding CobW3 resulted in a strongly decreased cobalt content that was at a level of approximately 4,000 Co ions per cell, compared with 20,000 Co ions per cell in the parental strain, AE104 (Table 3). A mutant lacking CobW2 had 13,000 Co ions per cell after growth under the same conditions. A ∆*cobW2* ∆*cobW3* double null mutant did not decrease the Co content any further than what was measured in the ∆*cobW3* single null mutant (Table 3). When 1 µM Co(II) was added to the growth medium, the parental strain increased its cellular cobalt content only slightly. By contrast, compared with the non-amended medium, the three ∆*cobW* mutants increased their intracellular cobalt content between 1.8-fold to 4.5-fold (Table 3). When 5 µM Co(II) was added to the medium, all four strains attained similar cobalt levels of between 39,000 for the double null mutant and 55,000 Co ions per cell for the parental strain.

Deletion of ∆*zupT* resulted in a decreased cobalt content in the cells of 36%, and this decreased number of cobalt ions per cell was not affected by additional deletion of either *cobW2, cobW3,* or both genes together (Table 3). All four ∆*zupT* strains had a higher Co ion content in this medium than the AE104 ∆*cobW3* mutant. When 1 µM or 5 µM Co(II) was added, all four ∆*zupT* mutants accumulated similar amounts of cobalt, which were always higher in the ∆*zupT* mutants than in the strains that had a native *zupT* gene.

Thus, CobW3 proved to be essential for the accumulation of cobalt in cells cultivated in standard TMM without added cobalt and depended on the presence of ZupT for accumulation of the cation. The lack of CobW3 caused a stronger decrease in cobalt import in the presence of ZupT than in its absence. CobW3 and ZupT may be involved in a downregulation of cobalt import by other import systems, for example, the CorAs, when sufficient zinc is available. Alternatively or additionally, CobW3 and ZupT together may stimulate efflux of Co under these conditions. CobW2 played a minor role in the control of cobalt accumulation in these cells.

### CobW2 and CobW3 control the cellular flow-equilibrium of zinc in *C. metallidurans*

**Fig 2:**
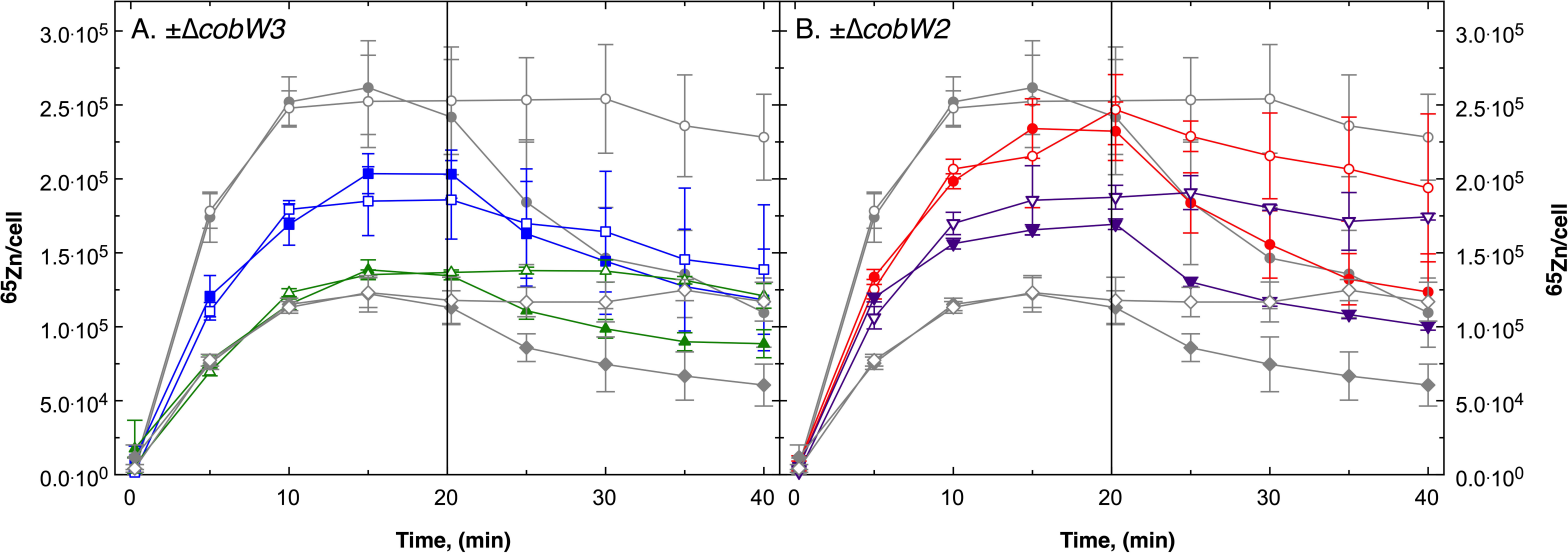
Pulse-chase experiment with *C. metallidurans* strains AE104, and its isogenic ∆*cobW2* and ∆*cobW3* mutants. Cells of strain AE104 (gray circles), ∆*zupT* (grey diamonds), ∆*cobW3* (blue squares), ∆c*obW2* (red circles), ∆*zupT* Δ*cobW2* (purple inverted triangles), and ∆*zupT* ∆*cobW3* (green triangles) were cultivated in low Zn and low Mg (0.1 mM Mg(II)) TMM. Panel A shows the strains without *cobW3*. Panel B those without *cobW2*. After cell-harvest, washing and suspension in uptake buffer, the cells were incubated in the presence of 1 µM ^65^Zn(II) in the pulse phase and chased at t = 20 min with 100 µM non-radioactive Zn(II) (black symbols), or remained unchased (open symbols). The data for strains AE104 and ∆*zupT* have already been published (18) and were obtained in the same experimental series as the other data.

Zinc homeostasis in *C. metallidurans* is based on the control of a flow-equilibrium between uptake and efflux reactions, in addition to an effect caused by the metal-binding cytoplasmic components glutathione and polyphosphate (18). Pulse-chase experiments were performed to investigate how CobW2 or CobW3 affects this flow-equilibrium of zinc. Cells were cultivated in TMM adjusted to 200 nM Zn(II) (ambient Zn, aZn), low zinc (lZn), and metal-starvation medium (lZn_lMg), which is low zinc TMM with 100 µM instead of 1 mM Mg(II) (Table S1) to the exponential phase of growth. Harvest and washed cells were then loaded for 20 min with 1 µM radioactive ^65^Zn and subsequently chased with 100 µM non-radioactive zinc. The cellular ^65^Zn content of the cells was measured as described (18), with a control that was not chased. The results obtained for AE104 and ∆*zupT* results have already been published (18) but were obtained in the same experimental series as the data for the ∆*cobW* mutants, and these data are shown again for reference (Fig. 2; Fig. S2 to S4). From the pulse-chase results, the initial uptake rate of ^65^Zn (v_up_), the cellular zinc content after the uptake period C_20_, the ratio of the extrapolated maximum zinc content of the uptake period C_max_ divided by C_20_, the initial efflux rates v_eff_ of the chase period in the chased and non-chased control cells, and the ratio of the zinc contents at 40 min C_40_ divided by C_20_ were calculated (Table 4).

**TABLE 4:** Summary of the ^65^Zn pulse-chase experiments

Medium	Uptake (pulse)			Chase		Pulse continued (control)	
Strains	v_up_ (s^−1^) , % AE104	C_20_, 1000 Zn/cell	C_max_/C_20_	v_eff_ (s^−1^), % AE104	C_40_/C_20_	v_eff_ (s^−1^), % AE104	C_40_/C_20_
Ambient Zn							
AE104*^a^*	162 ± 25; 100% ± 15%	96 ± 7; 100% ± 7%	2.20	112.3 ± 1.0; 100.0% ± 0.9%	37.2% ± 6.7%	−14.4 ± −0.2; −12.8% ± −0.2%	127.0% ± 8.8%
∆*zupT^a^*	94 ± 7; 58% ± 4%	64 ± 4; 66% ± 4%	2.67	38.8 ± 0.3; 34.5% ± 0.2%	46.4% ± 12.7%	2.1 ± 0.0; 1.9% ± 0.0%	102.5% ± 2.5%
∆*cobW2*	111 ± 4; 69% ± 3%	73 ± 2; 75% ± 2%	2.43	32.2 ± 0.0; 28.6% ± 0.0%	57.2% ± 10.0%	9.3 ± 0.0; 8.3% ± 0.0%	86.3% ± 17.9%
∆*cobW3*	90 ± 1; 56% ± 0%	54 ± 5; 56% ± 5%	1.99	23.7 ± 0.2; 21.1% ± 0.2%	61.9% ± 11.0%	−4.8 ± 0.0; −4.3% ± 0.0%	120.3% ± 21.6%
∆*zupT* ∆*cobW2*	96 ± 3; 59% ± 2%	83 ± 4; 87% ± 4%	4.66	15.5 ± 0.0; 13.8% ± 0.0%	82.2% ± 4.4%	−11.1 ± 0.0; −9.9% ± 0.0%	115.2% ± 8.4%
∆*zupT* ∆*cobW3*	94 ± 4; 58% ± 2%	49 ± 8; 51% ± 8%	1.73	3.2 ± 0.0; 2.8% ± 0.0%	99.6% ± 37.6%	−4.0 ± 0.0; −3.6% ± 0.0%	99.9% ± 53.5%
Low zinc							
AE104*^a^*	227 ± 17; 100% ± 8%	92 ± 3; 100% ± 3%	1.62	125.4 ± 1.3; 100.0% ± 1.0%	45.2% ± 16.6%	−11.2 ± −0.1; −8.9% ± −0.1%	128.1% ± 10.4%
∆*zupT^a^*	67 ± 1; 30% ± 0%	63 ± 4; 68% ± 4%	7.44	15.4 ± 0.1; 12.2% ± 0.0%	67.7% ± 2.1%	−6.7 ± 0.0; −5.3% ± 0.0%	118.8% ± 12.9%
∆*cobW2*	194 ± 34; 86% ± 15%	114 ± 6; 123% ± 6%	1.77	37.6 ± 0.1; 30.0% ± 0.1%	60.2% ± 10.0%	−11.3 ± −0.1; −9.0% ± 0.0%	109.2% ± 23.1%
∆*cobW3*	113 ± 28; 50% ± 12%	66 ± 5; 72% ± 6%	2.42	24.1 ± 0.0; 19.2% ± 0.0%	71.0% ± 10.5%	−4.8 ± 0.0; −3.8% ± 0.0%	104.0% ± 52.5%
∆*zupT* ∆*cobW2*	97 ± 2; 43% ± 1%	73 ± 4; 79% ± 4%	3.04	13.4 ± 0.0; 10.6% ± 0.0%	85.1% ± 12.3%	1.3 ± 0.0; 1.1% ± 0.0%	96.1% ± 25.3%
∆*zupT* ∆*cobW3*	104 ± 13; 46% ± 6%	67 ± 5; 73% ± 6%	2.41	7.1 ± 0.1; 5.6% ± 0.1%	93.2% ± 11.7%	−13.3 ± −0.1; −10.6% ± 0.0%	134.3% ± 33.4%
Low Zn and Mg							
AE104*^a^*	1147 ± 351; 100% ± 31%	272 ± 28; 100% ± 10%	1.45	314.4 ± 3.0; 100.0% ± 0.9%	40.2% ± 8.6%	27.1 ± 0.1; 8.6% ± 0.0%	83.8% ± 10.7%
∆*zupT^a^*	509 ± 10; 44% ± 1%	115 ± 4; 42% ± 1%	1.38	53.3 ± 0.2; 16.9% ± 0.1%	52.5% ± 12.3%	−2.1 ± 0.0; −0.7% ± 0.0%	101.4% ± 11.4%
∆*cobW2*	695 ± 72; 61% ± 6%	240 ± 10; 88% ± 4%	1.49	119.0 ± 0.4; 37.9% ± 0.1%	51.6% ± 10.8%	48.2 ± 0.0; 15.3% ± 0.0%	81.0% ± 20.9%
∆*cobW3*	677 ± 45; 59% ± 4%	195 ± 12; 71% ± 5%	1.42	86.9 ± 0.3; 27.6% ± 0.1%	60.8% ± 17.7%	46.3 ± 0.1; 14.7% ± 0.0%	71.3% ± 22.5%
∆*zupT* ∆*cobW2*	788 ± 291; 69% ± 25%	178 ± 13; 66% ± 5%	1.36	65.2 ± 0.3; 20.7% ± 0.1%	56.2% ± 1.5%	16.3 ± 0.0; 5.2% ± 0.0%	97.7% ± 1.2%
∆*zupT* ∆*cobW3*	382 ± 64; 33% ± 6%	136 ± 2; 50% ± 1%	1.63	44.7 ± 0.2; 14.2% ± 0.1%	65.3% ± 7.0%	14.1 ± 0.0; 4.5% ± 0.0%	89.2% ± 6.1%

^a^ Data obtained in the same experimental series and already published (18).

Fully zinc-replete cells of the ∆*zupT*, ∆*cobW2,* ∆*cobW3*, ∆*zupT* ∆*cobW2,* and ∆*zupT* ∆*cobW3* mutants cultivated in aZn-TMM showed insignificant differences between their respective pulse-chase curves (Fig. S2 and S3); however, all mutants accumulated a lower amount of ^65^Zn than the parent AE104 during the uptake phase, that is, after 20 min (Table 4). Although the parent was fully zinc-saturated after 20 min with 96,000 ^65^Zn imported, the respective value was only 75% for ∆*cobW2*, 66% for (∆*zupT),* and 50% for (∆*cobW3,* ∆*zupT* ∆*cobW3*). The lower zinc contents of the mutant cells led to a decrease of the initial efflux rate in the subsequent chase period, even as low as only 2.8% of the parental value for the ∆*zupT* ∆*cobW3* double null mutant.

Under zinc-starvation conditions (low zinc medium), the pulse-chase curves of the mutant cells were also not significantly different from each other but were different from those of the parent (Fig. S4). The ∆*cobW2* mutant was not different from AE104 with respect to the initial uptake velocity and ^65^Zn content C_20_ after the pulse period. Also, the ∆*zupT* ∆*cobW2* double mutant accumulated more zinc than the ∆*zupT* mutant and with a higher initial uptake rate (Table 4). Nevertheless, the initial net efflux rate v_eff_ decreased in all mutants, including the ∆*cobW2* strain, in comparison to AE104, and the rate in ∆*zupT* ∆*cobW3* mutant was reduced to 5.6% of the parental value.

Following cultivation under metal-starvation (for Zn, Co, Mg) (lZn_lMg metal-starvation TMM), the parent reached a maximum zinc content after only 15 min during the uptake period and even the non-chased control exported ^65^Zn after this period, whereas the ∆*zupT* mutant reached a level of zinc that was 60% lower, and the mutant continued to import zinc into un-chased control cells (Fig. 2; Table 4 (18)). Introduction of a *cobW3* deletion into this strain had a stronger negative effect than introduction of a ∆*cobW2* mutation (Fig. 2). The pulse-chase curves of the ∆*zupT* ∆*cobW3* double null mutant (Fig. 2A, green triangles) was similar to that of the ∆*zupT* single mutant, whereas that of the ∆*cobW3* mutant (Fig. 2A, blue squares) lay between the ∆*zupT* mutant and the parental strain. The curve of the ∆*zupT* ∆*cobW2* double null mutant lay between that of the ∆*zupT* mutant and the parental strain and that of the ∆*cobW2* mutant lay between that of the ∆*zupT* ∆*cobW2* double mutant and its parent (Fig. 2B). All mutants accumulated ^65^Zn with a lower initial uptake rate than AE104 and accumulated lower amounts of zinc after 20 min, except for the ∆*cobW2* mutant (Table 4). All mutants also exported ^65^Zn with a lower net efflux rate than the parent, with rates between 38% of the parental value in the ∆*cobW2* single mutant and as low as 14% in the ∆*zupT* ∆*cobW3* double null mutant (Table 4).

ZupT, in cooperation with CobW2 and CobW3, influenced the flow-equilibrium of zinc. The effect of a lack of CobW2 was smaller compared with when the gene encoding CobW3 was deleted. In contrast, the effect on the flow-equilibrium was stronger in zinc-replete cells than in zinc- or metal-starved cells. Without these CobW proteins, the initial uptake rates, zinc contents after 20 min incubation with 1 µM Zn(II), and initial net efflux rates were lower compared with the parent. As indicated by the results from metal-starved cells, lack of CobW2 and CobW3 impacted zinc import by ZupT, as well as on other uptake systems (Fig. 2), with a ∆*cobW2* deletion even increasing zinc import in a ∆*zupT* background (Fig. 2B). Thus, these three proteins together seem to control zinc homeostasis in *C. metallidurans* (Table 4), and they also influence the cobalt content of the cells (Table 3).

### ZupT, CobW2, and CobW3 control the cellular zinc pools

The pulse-chase experiments with radioactive ^65^Zn were performed in parallel with experiments using isotope-enriched stable ^67^Zn solutions (18). This allowed us to differentiate between a zinc pool, ZP1, containing zinc with the natural isotope composition and a ZP2 stemming from the isotope-enriched ^67^Zn solution. The cells were cultivated in ambient zinc (aZn), zinc-starvation (lZn), and zinc-magnesium-, metal-starvation (lZn_lMg) medium with zinc in the natural isotope composition. Incubation was for 20 min with 1 µM ^67^Zn(II) and chased for an additional 20 min with 100 µM zinc, again with the natural isotope composition. In this way, ZP2 represented the zinc imported during the uptake phase, and ZP1 represented zinc that was initially present in the cells and also at the end of the experiment.

As with radioactive zinc, deletion of the genes for ZupT and both CobWs decreased the initial zinc content in ZP1 in zinc-replete cells (Table 5). The strongest effect was measured in the ∆*zupT* ∆*cobW3* double null mutant. Incubation of cells with 1 µM ^67^Zn resulted in zinc appearing in ZP2, a decrease of zinc in ZP1 but insignificant change in the overall zinc content, that is, ZP1 + ZP2. Zinc ions that were initially present in cells were exchanged against incoming zinc ions, and ZupT, CobW2, and CobW3 were not required for this turnover of zinc (Table 5). During the subsequent chase, zinc was mainly exported or exchanged from ZP2, despite the overall zinc content increasing in the cells. In zinc-replete cells, deletion of either of the three genes *zupT, cobW2,* or *cobW3* individually resulted in a decreased Co and Ni content of the cells (Table S5).

**TABLE 5:** Summary of the experiments with stable ^67^Zn that accompanied the pulse-chase experiments with radioactive ^65^Zn^*b*^

Strains	Initial 10^3^ Zn			10^3^ Zn after pulse			10^3^ Zn after chase (0.1 mM)		
Medium	ZP1	ZP2; %ZPt	ZP1 +ZP2	ZP1	ZP2; %ZPt	ZP1 +ZP2	ZP1	ZP2; %ZPt	ZP1 +ZP2
Ambient Zn									
AE104^*a*^	103 ± 9	0.0 ± 0.2; 0.0%	103 ± 9	76.6 ± 1.8	27.4 ± 2.2; 26.4%	104 ± 4	240 ± 20	6.1 ± 0.8; 2.5%	246 ± 21
∆*zupT^a^*	42.8 ± 1.8	<0	42.6 ± 1.7	37.8 ± 2.1	20.5 ± 1.2; 35%	58.3 ± 3.3	226 ± 15	4.1 ± −0.1; 2%	230 ± 14
∆*cobW2*	73.9 ± 2.6	<0	73.6 ± 2.6	60.9 ± 0.6	21.8 ± 1.3; 26%	82.8 ± 1.9	212 ± 26	3.7 ± 0.3; 2%	215 ± 26
∆*cobW3*	77.1 ± 2.1	<0	76.8 ± 2.1	64.8 ± 1.6	21.9 ± 1.3; 25%	86.7 ± 2.9	307 ± 44	3.8 ± 0.2; 1%	311 ± 44
∆*zupT* ∆*cobW2*	57.8 ± 8.8	<0	57.6 ± 8.8	46.9 ± 6.5	20.8 ± 0.4; 31%	67.7 ± 6.9	238 ± 15	4.3 ± 0.1; 2%	243 ± 15
∆*zupT* ∆*cobW3*	40.3 ± 0.8	<0	40.2 ± 0.8	36.1 ± 1.1	15.1 ± 0.7; 29%	51.1 ± 1.8	168 ± 8	4.1 ± 0.0; 2%	172 ± 7
Low Zn									
AE104^*a*^	11.0 ± 1.9	0.0 ± 0.0; 0.1%	11.1 ± 1.9	6.7 ± 0.7	79.4 ± 5.3; 92.3%	86.1 ± 6.0	181 ± 17	24.6 ± 1.4; 12.0%	206 ± 19
∆*zupT^a^*	43.2 ± 1.4	<0	43.0 ± 1.4	37.0 ± 0.8	30.8 ± 2.5; 45%	67.8 ± 3.3	190 ± 8	6.9 ± 0.2; 4%	197 ± 8
∆*cobW2*	8.9 ± 4.5	0.0 ± 0.0; 0%	8.9 ± 4.5	9.5 ± 3.9	58.4 ± 1.4; 86%	67.9 ± 5.3	180 ± 3	15.5 ± 0.8; 8%	196 ± 4
∆*cobW3*	8.9 ± 4.6	0.0 ± 0.0; 0%	8.9 ± 4.5	15.4 ± 6.6	42.8 ± 1.7; 74%	58.2 ± 8.3	166 ± 9	15.1 ± 0.3; 8%	182 ± 10
∆*zupT* ∆*cobW2*	5.3 ± 0.5	0.0 ± 0.0; 1%	5.3 ± 0.5	8.3 ± 3.1	53.5 ± 0.7; 87%	61.8 ± 3.8	161 ± 3	14.1 ± 0.3; 8%	176 ± 4
∆*zupT* ∆*cobW3*	10.1 ± 6.4	0.0 ± −0.1; 0%	10.1 ± 6.3	8.3 ± 5.5	12.5 ± 0.2; 60%	20.7 ± 5.7	138 ± 13	4.7 ± −0.1; 3%	143 ± 13
Low Zn and Mg									
AE104^*a*^	29.5 ± 10.2	0.5 ± 0.0; 1.6%	30.0 ± 10.1	6.3 ± 1.3	85.0 ± 3.9; 93.1%	91.3 ± 5.3	201 ± 33	25.4 ± 2.0; 11.2%	226 ± 35
∆*zupT^a^*	8.3 ± 0.8	<0	8.3 ± 0.8	8.0 ± 0.3	57.4 ± 3.0; 88%	65.4 ± 3.3	169 ± 22	13.9 ± 0.3; 8%	183 ± 22
∆*cobW2*	21.4 ± 10.4	<0	21.4 ± 10.3	20.0 ± 10.1	56.3 ± 9.0; 74%	76.3 ± 19.1	175 ± 14	14.6 ± 3.0; 8%	190 ± 17
∆*cobW3*	11.9 ± 1.3	7.0 ± 9.4; 37%	18.9 ± 10.8	10.2 ± 1.2	40.4 ± 2.6; 80%	50.6 ± 3.8	166 ± 22	14.9 ± 0.1; 8%	181 ± 23
∆*zupT* ∆*cobW2*	12.9 ± 4.0	<0	12.9 ± 4.0	11.4 ± 3.0	57.6 ± 2.0; 83%	69.1 ± 5.0	174 ± 6	13.8 ± 0.8; 7%	188 ± 7
∆*zupT* ∆*cobW3*	10.4 ± 0.4	<0	10.4 ± 0.4	8.4 ± 0.5	14.2 ± 0.9; 63%	22.6 ± 1.4	171 ± 7	5.5 ± 0.0; 3%	177 ± 7

^*a*^ Data obtained in the same experimental series and already published (18).

^*b*^ The cells of the indicated *C. metallidurans* mutants were incubated in Tris-buffered mineral salts medium adjusted to 200 nM Zn(II) (ambient zinc); the same medium without trace element solution SL6 and 0.1 mM Mg(II) instead of 1 mM Mg(II) (low Zinc and Mg), or TMM medium without SL6 but with 1 mM Mg(II) (low Zn). Zinc coming from SL6 or contaminations was in the natural isotope composition. These cells were incubated with 1 µM enriched stable ^67^Zn for 20 min (pulse) and subsequently chased with 100 µM Zn(II) (or 1 mM when indicated) with the natural isotope composition. The zinc pools ZP1 and ZP2 were calculated from the ICP-MS measurements and ZPt = ZP1+ZP2 was determined.

In low-zinc and in low-zinc, low-magnesium media, the cellular zinc content of the cells was lower compared with zinc-replete (aZn) cells, as expected. The high zinc content in the cells of the ∆*zupT* strain after growth in low-zinc medium was twice as high as in all other comparable experimental series and was not considered. Compared with zinc-replete cells, no zinc was exported from ZP1 during the uptake phase in zinc-starved cells, and more zinc remained in ZP2 during the subsequent chase. The exception was the parent in low-zinc, low-magnesium medium. This strain decreased the zinc content in ZP1 during the uptake phase, but its mutant derivatives did not. The zinc content in ZP2 was particularly low in cells of the zinc-starved ∆*zupT* ∆*cobW3* double null mutant compared with the other cells, both before and after the chase (Table 5).

These results allow us to propose that the three proteins ZupT, CobW2, and CobW3 are required to adjust the cellular zinc pools under all conditions examined. The absence of ZupT and CobW3 results in decreased uptake of zinc, whereas efflux of zinc during the chase period was barely affected (Table 5, clearance of ZP2 during the chase period).

### ZupT, CobW2, and CobW3 affect Co, Cd, and EDTA resistance

Single or double deletions of *cobW2* and/or *cobW3* in the *C. metallidurans* parental strain AE104 did not decrease zinc or cobalt resistance, but cadmium resistance was decreased (Table 6). In the double mutant resistance to the metal cation-chelator, ethylenediaminetetraacetate (EDTA) was also decreased (Table 6). This decrease in resistance was even stronger in the ∆*zupT* background. Here, cobalt resistance was reduced to 43% in the ∆*zupT* mutant and 8% in the ∆*zupT* ∆*cobW3* double null mutant, whereas a ∆*cobW2* deletion did not decrease any further the low cobalt resistance level of the ∆*zupT* mutant. The pattern of EDTA resistance in the ∆*zupT* mutant was similar to that for its cobalt resistance and accounted for a reduction in resistance to 45% in ∆*zupT* compared with the parent, and a further minor decrease in the ∆*zupT* ∆*cobW2* double mutant, but resistance was decreased to 10% of the ∆*zupT* resistance level in the ∆*zupT ∆cobW3* mutant (Table 6).

**TABLE 6:** IC_50_ values of mutant strains^*a*^

Strain	Zn (µM)	Co (µM)	Cd (µM)	EDTA (µM)
AE104	114.2 ± 15.6 (1.00; 0.0)	193 ± 9 (1.00; 0.0)	174 ± 10 (1.00; 0.0)	1,652 ± 58 (1.00; 0.0)
Δ*cobW3*	94.3 ± 2.8 (0.83; 1.1)	176 ± 14 (0.91; 0.7)	**114 ± 15 (0.65; 2.4)**	1,353 ± 180 (0.82; 1.3)
Δ*cobW2::dis*	96.0 ± 7.4 (0.84; 0.8)	182 ± 15 (0.94; 0.5)	**112 ± 5 (0.65; 4.1)**	1,185 ± 156 (0.72; 2.2)
Δ*cobW3* Δ*cobW2::dis*	92.4 ± 4.9 (0.81; 1.1)	174 ± 19 (0.90; 0.6)	**87.5 ± 12.3 (0.50; 3.9)**	**975 ± 276 (0.59; 2.0)**
				
Δ*zupT*	86.8 ± 3.8 (0.76; 1.4)	**83.8 ± 4.8 (0.43; 7.7)**	**39.1 ± 3.8 (0.23; 9.9)**	**739 ± 154 (0.45; 4.3)**
Δ*zupT* Δ*cobW3*	65.0 ± 5.6 (0.75; 2.3)	**6.6 ± 0.1 (0.08; 15.8)**	**4.8 ± 1.2 (0.12; 6.9)**	**75.5 ± 19 (0.10; 3.8)**
Δ*zupT* Δ*cobW2::dis*	108.2 ± 1.2 (1.25; 4.3)	93.0 ± 9.0 (1.11; 0.7)	**9.0 ± 0.2 (0.23; 7.5)**	548 ± 96 (0.74; 0.8)
Δ*zupT* Δ*cobW3* Δ*cobW2::dis*	78.4 ± 1.7 (0.90; 1.5)	86.1 ± 3.4 (1.03; 0.3)	**4.3 ± 0.0 (0.11; 9.2)**	523 ± 230 (0.71; 0.6)
				
Δ*dmeF*	106.6 ± 8.7 (0.93; 0.3)	**5.5 ± 0.7 (0.03; 18.7)**	149 ± 25 (0.86; 0.7)	1,343 ± 348 (0.81; 0.8)
Δ*dmeF* Δ*cobW3*	97.6 ± 2.0 (0.92; 0.8)	7.9 ± 0.6 (1.44; 1.8)	132 ± 17 (0.89; 0.4)	1,921 ± 222 (1.43; 1.0)
Δ*dmeF* Δ*cobW2::dis*	98.5 ± 2.1 (0.92; 0.8)	5.2 ± 0.4 (0.95; 0.3)	113 ± 6 (0.76; 1.1)	1,809 ± 164 (1.35; 0.9)
Δ*dmeF* Δ*cobW3* Δ*cobW2::dis*	97.6 ± 2.6 (0.92; 0.8)	6.2 ± 0.3 (1.13; 0.7)	104 ± 11 (0.70; 1.2)	1,721 ± 268 (1.28; 0.6)
				
Δ*dmeF* Δ*zupT*	78.0 ± 1.7 (0.68; 2.10)	**2.1 ± 0.1 (0.01; 20.1)**	**40.5 ± 1.7 (0.23; 11.5)**	1,263 ± 17 (0.76; 5.19)
Δ*dmeF* Δ*zupT* Δ*cobW3*	77.0 ± 2.3 (0.99; 0.26)	2.6 ± 0.2 (1.22; 1.44)	**72.9 ± 0.9 (1.80; 12.5)**	1,578 ± 34 (1.25; 6.20)
Δ*dmeF* Δ*zupT* Δ*cobW2::dis*	73.6 ± 3.4 (0.94; 0.87)	2.3 ± 0.1 (1.06; 0.47)	**12.9 ± 2.2 (0.32; 7.05)**	1,017 ± 39 (0.81; 4.45)
Δ*dmeF* Δ*zupT* Δ*cobW3* Δ*cobW2::dis*	75.5 ± 1.4 (0.97; 0.81)	2.5 ± 0.0 (1.18; 1.96)	**13.8 ± 4.5 (0.34; 4.30)**	**686 ± 93 (0.54; 5.28)**

^*a*^ Standard TMM. Bold-faced if [(Q > 1.5 OR Q < 0.67) AND D > 1], meaning the ratios of two values are larger than 1.5 and the deviation bars do not overlap. Comparison of the ∆*dmeF* and the ∆*zupT* mutants to AE104 and of the ∆*cobW* mutants to their respective parent.

The effect of these gene deletions in reducing resistance was even stronger with respect to cadmium resistance, where a decrease down to 23% of the parental level in the ∆*zupT* mutant was observed. A further decrease to 23% compared with the level in the ∆*zupT* mutant was observed for the ∆*zupT* ∆*cobW2* double mutant, and an even stronger decrease down to 12% was measured for the ∆*zupT* ∆*cobW3* double mutant (Table 6). Additional deletion of *cobW2* increased the low cobalt and EDTA resistance level of the ∆*zupT* ∆*cobW3* double mutant to approximately the level of the ∆*zupT* mutant. In contrast, cadmium resistance was not affected by the additional deletion of *cobW2* in the ∆*zupT ∆cobW3* strain.

These data suggest that CobW2 and CobW3 cooperate to mediate full cadmium resistance in the parental strain and in its ∆*zupT* mutant derivative. On the other hand, the absence of CobW3 decreased cobalt and EDTA resistance in the ∆*zupT* strain, but not in the parent. This strong effect of a ∆*cobW3* deletion in the ∆*zupT* strain with respect to the Co and EDTA resistance, but not the Cd resistance, was reversed again by further deletion of *cobW2*, indicating that CobW2 mediated the low Co and EDTA resistance level in the ∆*zupT* ∆*cobW3* double mutant.

### DmeF is an important valve for the release of surplus cobalt ions

**Fig 3:**
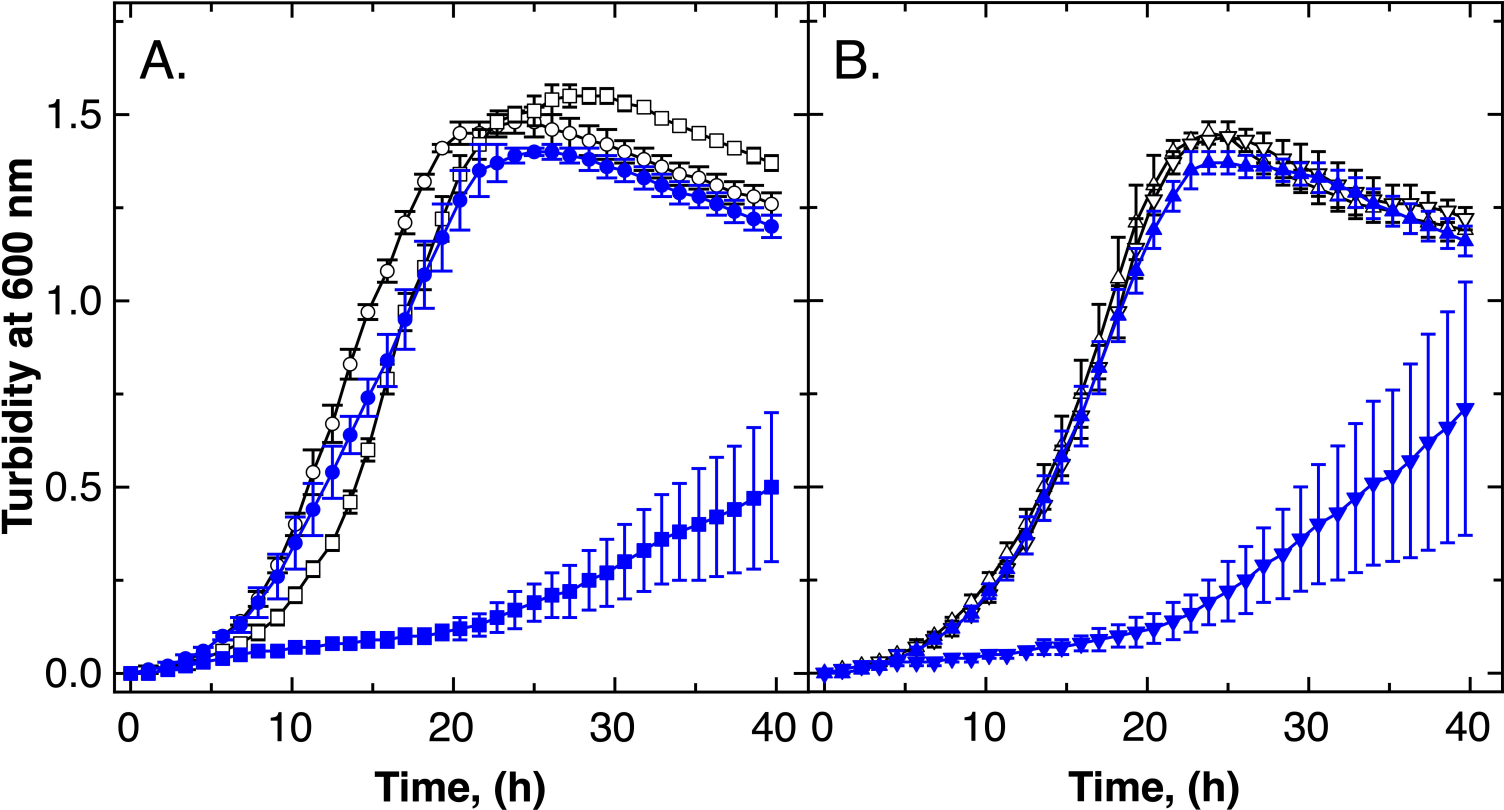
DmeF mediates cobalt resistance in *C. metallidurans*. Panel A shows time-dependent growth of the parental strain, AE104 (circles), and an isogenic ∆*dmeF* mutant (squares). Panel B shows data for a ∆*fieF* (triangles) mutant and a ∆*dmeF* Δ*fieF* (inverted triangles) cultivated with (blue closed symbols) or without (black open symbols) 5 µM co(II) in standard TMM. Data for three biological repeats with standard deviations are shown.

The three proteins ZupT, CobW2, and CobW3 control accumulation of cobalt in zinc-starved cells and are involved in resistance to Co, Cd, and EDTA. The main cadmium exporters in *C. metallidurans* are the P_IB2_-type ATPases ZntA and CadA (15), whereas the CDF protein DmeF is required for cobalt export (15, 16). A ∆*dmeF* mutant showed impaired growth in the presence of 5 µM Co(II) in mZn medium (Fig. 3). The IC_50_ of the mutant was 5.5 µM Co(II) compared with 193 µM in the parental strain, AE104 (Table 6). Resistance to zinc, cadmium, and EDTA was not influenced in the mutant. The CDF protein FieF in AE104 is required for resistance to iron (16, 45). Growth of a ∆*fieF* mutant was only slightly impaired compared with AE104, whereas a ∆*dmeF* ∆*fieF* double mutant had a growth phenotype similar to the ∆*dmeF* single mutant (Fig. 3B). Additional deletion of the genes *cobW2, cobW3,* or both in the ∆*dmeF* strain had limited influence on resistance to Zn, Co, Cd, or EDTA (Table 6).

The cobalt content of cells of the ∆*dmeF* mutant cultivated in standard zinc medium was identical to that of the parent, AE104 (Table 3). When 1 µM or 5 µM Co was added, the level of the cation increased 3.5-fold and 6.2-fold, respectively, to a final level of 130,000 Co per cell, whereas the zinc content of the cells was not influenced (Table S4). These data confirm that DmeF is the major cobalt efflux system of *C. metallidurans* AE104. The CobWs did not mediate any level of cobalt resistance in the absence of DmeF (Table 6).

### DmeF supports the function of the ZupT, CobW2, and CobW3 network

**Fig 4:**
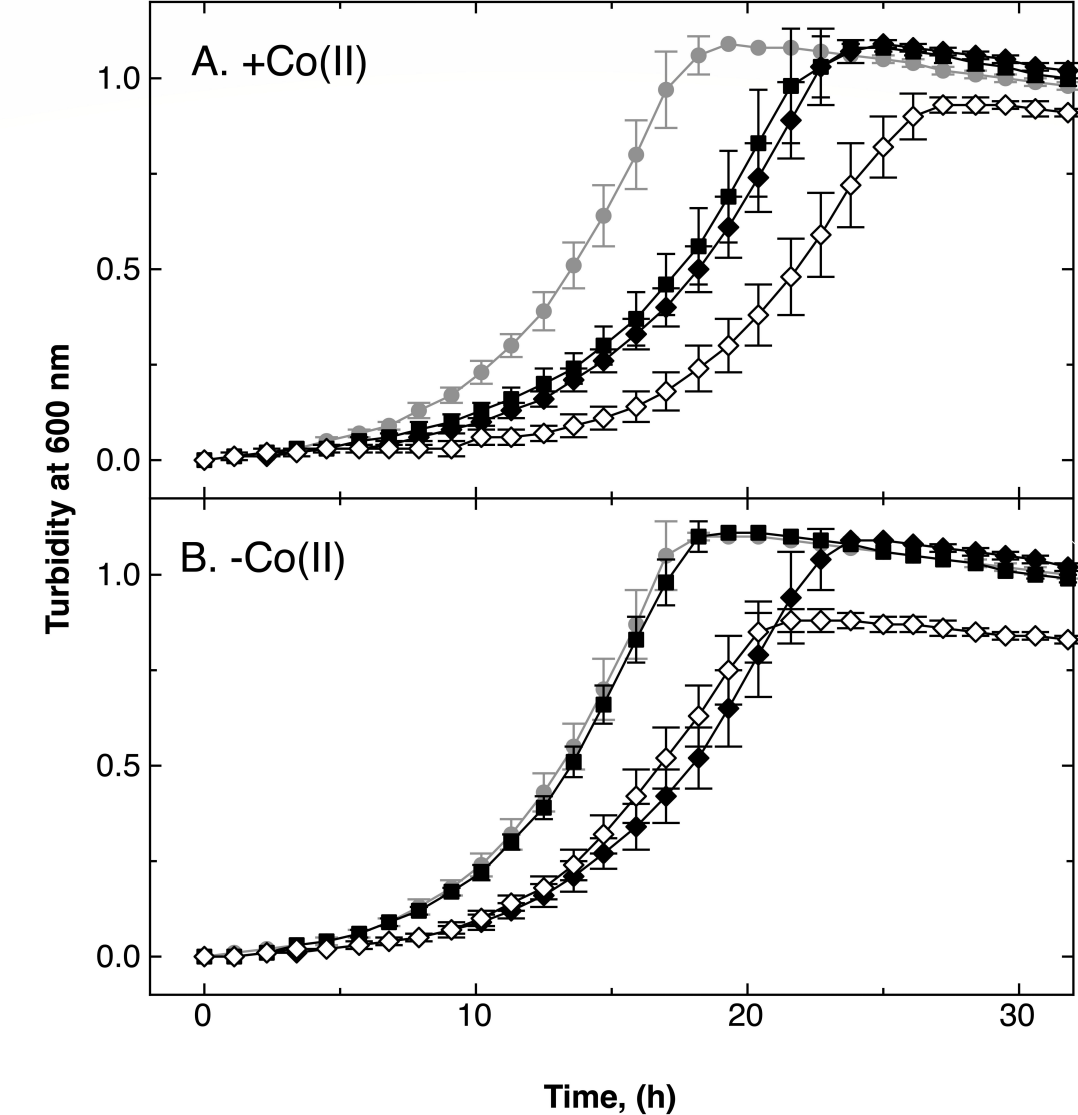
Effect of Co(II) on a Δ*dmeF* ∆*zupT* double null mutant. Time-dependent growth curves of strains ∆*zupT* (closed diamonds), ∆*dmeF* (squares), and ∆*dmeF* Δ*zupT* (open diamonds) in standard TMM medium without (panel B) or with (panel A) addition of 1 µM co(II) are shown. Growth of the parental strain AE104 (closed circles) is shown in gray for reference. Data for three biological repeats with standard deviations were shown.

All mutants up to the quadruple mutant ∆*dmeF* ∆*zupT* ∆*cobW3* ∆*cobW2::dis* were constructed and characterized. Cobalt resistance of the ∆*zupT* mutant was 43% of the level of the parental strain, AE104, and that of the ∆*dmeF* mutant only 3% of the parent (Table 6). The IC_50_ for Co(II) decreased to 1% of the parental level in the ∆*dmeF* ∆*zupT* double deletion mutant (Table 6). In the presence of 1 µM Co(II), the double mutant grew more slowly than AE104, whereas both single mutants had similar growth rates that ranged between that of the double mutant and that of AE104 (Fig. 4A). In the absence of added Co(II), the ∆*dmeF* mutant grew like its parent, the ∆*zupT* mutant grew more slowly, and the double mutant’s growth rate was similar to that of the ∆*zupT* mutant, albeit with a lower growth yield (Fig. 4B). Both transport systems, the ZupT uptake, and the DmeF efflux systems, thus appear to cooperate to mediate cobalt resistance and also to allow the maximum growth yield in the absence of added Co(II) to be attained.

**Fig 5:**
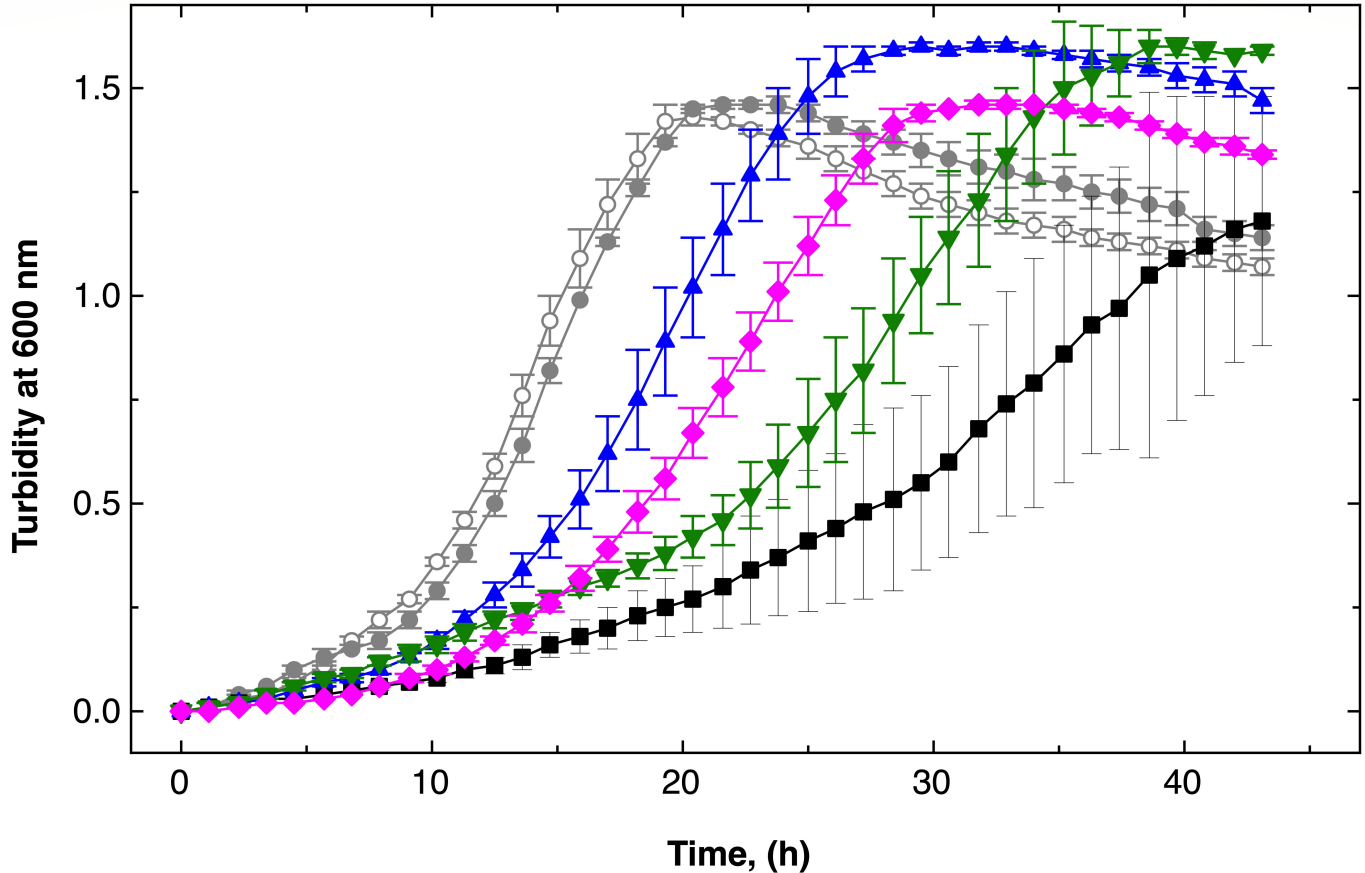
Strains lacking CobW2 and CobW3 show impaired growth in a ∆*dmeF* mutant background. Time-dependent growth of strains ∆*dmeF* (black filled squares), ∆*dmeF* Δ*cobW3* (blue triangles), ∆*dmeF* Δ*cobW2::dis* (green inverted triangles), and the ∆*dmeF* Δ*cobW3* Δ*cobW2::dis* triple mutant (magenta diamonds) in standard TMM in the presence of 2.5 µM co(II). Growth of the parental strain AE104 with (closed circles) and without (open circles) 2.5 µM co(II) is shown in gray for reference. Three biological repeats were performed, and standard deviations are shown. Data for all strains and conditions tested are shown in Supplementary information.

Deletion of *cobW2* or *cobW3* in the ∆*dmeF* isogenic strain increased Co resistance in the case of the ∆*dmeF* ∆*cobW3* strain. The IC_50_ of the ∆*dmeF* ∆*cobW2* strain did not change, however, and the triple mutant exhibited an IC_50_ like that of the ∆*dmeF* strain (Table 6). This effect was more clearly visible in growth curves done in the presence of 2.5 µM Co(II) (Fig. 5). The ∆*cobW3* deletion (Fig. 5, blue triangles) almost restored growth to the parental level, whereas the ∆*cobW2* deletion was less efficient in counteracting the growth delay mediated by the ∆*dmeF* mutation (green inverted triangles). The triple mutant had a growth rate that was between those of the ∆*dmeF* ∆*cobW2* and ∆*dmeF* ∆*cobW3* (magenta diamonds) mutants. This influence on the growth rate was only visible in the ∆*dmeF* mutant background and only in the presence of Co(II) (Fig. S5). Zinc, cadmium, and EDTA resistances were unchanged (Table 6). This suggests that CobW2 and CobW3 were responsible for the growth defect of the ∆*dmeF* mutant in the presence of Co(II). Moreover, both CobW proteins interacted with each other, with loss of CobW3 causing a more severe growth deficiency compared with loss of CobW2.

**Fig 6:**
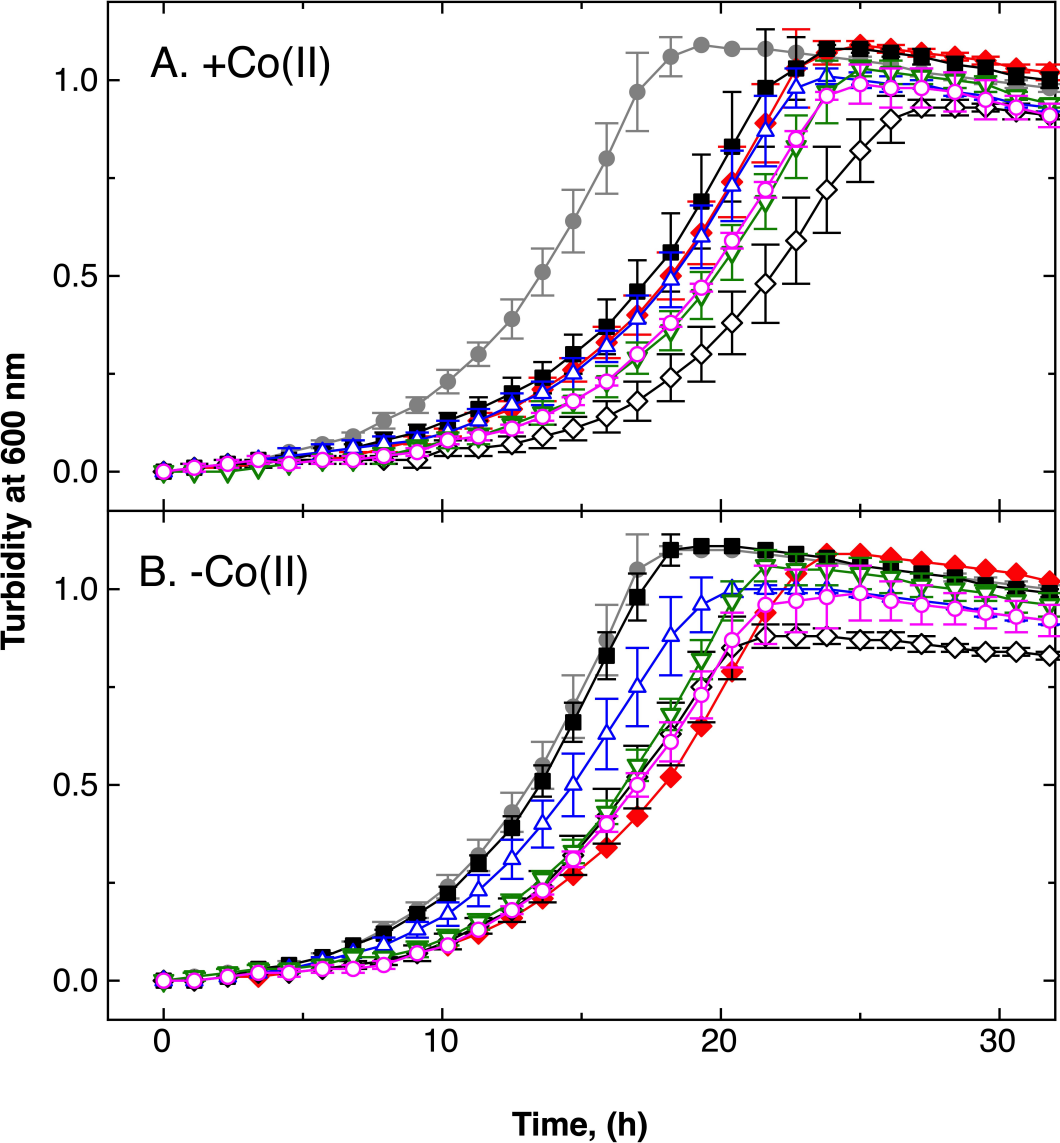
Effect of Co(II) on derivatives of the double deletion strain ∆*zupT* Δ*dmeF*. Time-dependent growth of strains ∆*zupT* (red filled diamonds), ∆*dmeF* (filled squares), ∆*dmeF* Δ*zupT* (open diamonds), ∆*dmeF* Δ*zupT* Δ*cobW2* (green open inverted triangles), ∆*dmeF* Δ*zupT* Δ*cobW3* (blue open triangles), and Δ*dmeF* Δ*zupT* Δ*cobW2* Δ*cobW3* (magenta open circles) in standard TMM medium without (panel B) or with (panel A) 1 µM co(II). Growth of the parent AE104 (closed circles) and is shown in gray for references. Three repeats, deviations indicated.

Introduction of a deletion in either the *cobW2* or *cobW3* genes in the ∆*dmeF* ∆*zupT* double mutant background also negatively affected growth in the presence or absence of 1 µM Co(II) (Fig. 6). In the presence of Co(II), the half-logarithmic plot shows that all growth curves were parallel with each other, except that of the parent, AE104 (Fig. S6). Compared with the parent in the presence of Co(II), the deletions affected the length of the lag-phase and the growth rate. Comparing the mutants with each other in the presence of Co(II) and in all comparisons in the absence of Co(II), only the length of the lag-phase was influenced.

When Co(II) was added, ∆*dmeF,* ∆*zupT*, and ∆*dmeF* ∆*zupT* ∆*cobW3* had the same growth delay, which was largest in the ∆*dmeF* ∆*zupT* double mutant and between those of the quadruple mutant and the ∆*dmeF* ∆*zupT* ∆*cobW2* triple mutant (Fig. 6; Fig. S6). As in the ∆*dmeF* mutant, the CobWs were also responsible for the growth delay of the ∆*dmeF* ∆*zupT* double mutant, but the difference in comparison to the ∆*dmeF* single mutant was that CobW2 no longer had any effect. In the absence of added Co(II), ∆*dmeF* mutant grew like the parent AE104, whereas most other mutants had growth phenotypes like the ∆*zupT* mutant, and only the ∆*dmeF* ∆*zupT* ∆*cobW3* strain had a growth phenotype between that of the ∆*zupT* and AE104. A decreased growth yield was only visible in the ∆*dmeF* ∆*zupT* double mutant, and therefore, this was also due to CobW2 or CobW3 (Fig. 6).

Introduction of a ∆*cobW3* mutation into the ∆*dmeF* ∆*zupT* double mutant increased Co resistance slightly, which was reminiscent of the phenotype of the ∆*dmeF* mutant; introducing the ∆*cobW2* allele had no effect (Table 6). Although the ∆*zupT* deletion decreased the cobalt content in standard TMM down to 36% of the parent level, deletion of *zupT* in the ∆*dmeF* mutant barely affected Co levels when compared with those of AE104 (Table 3). DmeF is therefore responsible for maintaining the low cobalt level in the ∆*zupT* strain. Moreover, although deletion of *cobW3* had only a small effect in the ∆*zupT* background, the mutation strongly decreased the cobalt content in the parental strain and its isogenic ∆*dmeF* mutant. However, the *cobW3* mutation had no effect in the ∆*dmeF* ∆*zupT* double null mutant background. Thus, ZupT was responsible for maintaining the low cobalt levels of the parental strain and in a ∆*dmeF* mutant. This means that DmeF, ZupT, and CobW3 cooperate to adjust the cobalt content of the cells in standard TMM.

These effects of the DmeF, ZupT, and CobW3 network on the cobalt level were similar in cells grown in the standard TMM with or without 1 µM Co(II) (Table 3), but the cobalt level increased strongly in the ∆*zupT* ∆*dmeF* ∆*cobW2* ∆*cobW3* quadruple mutant in the presence of 1 µM Co(II). Cobalt resistance of the quadruple mutant was about 2 µM (Table 6) and did not change when CobW2 or CobW3 were present. Neither CobW mediated some degree of cobalt resistance in the absence of ZupT and DmeF. In the presence of DmeF and the ∆*zupT* ∆*cobW2* ∆*cobW3* triple mutant, cobalt resistance increased 3-fold to about 6 µM (Table 6), so that ZupT caused a low degree of cobalt resistance, probably by mediating controlled uptake of Co(II). Again, presence of the CobWs in the ∆*dmeF* mutant background did not increase cobalt resistance, showing the importance of DmeF as central inner membrane efflux system for Co(II). Interestingly, cobalt resistance of the ∆*zupT* ∆*cobW3* double mutant was at the level of that of the ∆*dmeF* mutants and increased again, when *cobW2* was additionally deleted (Table 6). This indicated that CobW2 also has a function in cobalt homeostasis, which may be based on some interplay with the efflux system DmeF.

Although the EDTA resistance was strongly reduced in the ∆*zupT* mutant and even more so in the ∆*zupT* ∆*cobW3* double mutant, it was hardly changed in the ∆*dmeF* single mutant but was decreased in the ∆*dmeF* ∆*zupT* mutant down to 76% of the parental level. It increased again marginally (1.25-fold) when the *cobW3* gene was additionally deleted and was decreased to 81% of the ∆*dmeF* ∆*zupT* level by introduction of a ∆*cobW2* allele and was reduced further to 54% in the ∆*cobW3* ∆*cobW2* mutant. CobW2 and CobW3 synergistically increased EDTA resistance in the presence of ZupT and DmeF, had no effect when DmeF was absent from cells but ZupT was present, and acted antagonistically when either transport system was absent; CobW2 increased and CobW3 decreased resistance. The strongest effect on EDTA resistance was observed in the absence of ZupT but in the presence of DmeF with CobW3 increasing EDTA resistance 10-fold in a CobW2-dependent manner. This indicates that this CobW2-dependent decrease in EDTA resistance in the ∆*zupT* ∆*cobW3* mutant was also DmeF-dependent.

Although both CobWs contributed synergistically to Cd resistance in the presence of both transport systems, they had no effect in the ∆*dmeF* mutant. Deletion of ∆*cobW3* increased Cd resistance nearly 2-fold in the ∆*dmeF ∆zupT* double mutant while the same deletion decreased Cd resistance strongly in the ∆*zupT* strain. This effect was again CobW2-dependent. The IC_50_ for cadmium was similar in the quadruple mutant, the ∆*dmeF* ∆*zupT* ∆*cobW2* triple mutant, and the *∆zupT* ∆*cobW2* double mutant but was 2-fold higher than in the ∆*zupT ∆cobW3 ∆cobW2* triple and the ∆*zupT* ∆*cobW3* double mutants. DmeF was thus responsible for the strong decrease in cadmium resistance in the ∆*zupT* ∆*cobW3* double mutant (Table 6). This indicated that the ability to adjust the zinc and cobalt content through an interaction between ZupT, CobW2, CobW3, and DmeF is a prerequisite for the bacterium to adapt to metal starvation conditions and also for cadmium resistance. Zinc and cobalt homeostasis and cadmium resistance are linked processes in this bacterium with ZupT, CobW2, CobW3, and DmeF being the main actors in this multiple-ion homeostasis (Fig. S1).

## DISCUSSION

### The interplay between ZupT, CobW2, CobW3, and DmeF

A complicated interplay between ZupT with CobW2 and CobW3 (Fig. S1) affects the flow-equilibrium of Zn(II) in *C. metallidurans*, along with uptake of other metals, metal efflux systems, and the metal-binding components of the cytoplasm glutathione and polyphosphate (18). This maintains the number of Zn ions per cell at about 70,000 to 80,000 per cell, as previously published (8, 43). To accumulate this number of Zn ions per cell, a zinc content of the growth medium of 140 nM to 160 nM is needed, and indeed, at lower concentrations, all available Zn is accumulated by the cells (Table 2).

If the zinc content of the growth medium does not allow accumulation of 80,000 Zn ions per cell, *C. metallidurans* accumulates Co(II) (Fig. 1). This may allow us to metalate metal-promiscuous enzymes such as FolE_IB1 and FolE_IB2 to substitute Zn-dependent paralogs (25). ZupT and CobW3 are central to this process (Fig. 1; Tables 2 and 3), whereas CobW2 also contributes but in a minor way. The interplay of these three proteins is necessary for *C. metallidurans* to survive metal-starvation conditions, but it also contributes to cadmium and cobalt resistance (Table 3), with the Co efflux system DmeF (16) also required for full resistance to both metals. DmeF is as important for full cobalt resistance as ZupT (Fig. 4). CobW2 and CobW3 cause delayed growth in a ∆*dmeF* mutant in the presence of ZupT, so that all four proteins are necessary not only to mediate cobalt resistance but also to allow cobalt accumulation in response to zinc starvation.

Cd(II) interacts with all thiol compounds in the cytoplasm, which leads to protein denaturation, limiting levels of glutathione, release of iron from enzymes such as aconitase and subsequently to redox stress (46–49). The influence of ZupT, DmeF, and the CobW proteins in cadmium resistance (Table 6) also indicates that cadmium may disturb zinc homeostasis. Both metals belong to the same group of the periodic system of the elements and are thus chemically related. Disturbance of zinc homeostasis by cadmium has been noted before (50, 51). This would indicate that a primary function of ZupT, DmeF, and the two CobWs would be to protect zinc homeostasis against cadmium by filling-up the zinc pool with available zinc. Under zinc-replete conditions, cadmium cannot outcompete Zn(II) because Cd(II) is a soft metal ion that prefers Cys over His residues, and both metal ions have different ionic radii (52, 53). Only if insufficient Zn(II) is available to fill-up the zinc pool is Cd(II) able to outcompete Zn(II) (40). Filling up the zinc pool with cobalt instead may thus help prevent this toxic effect of Cd(II).

This could be one important reason to fill-up parts of the cellular zinc pool with Co(II) in the event of zinc-limitation. Moreover, metal-promiscuous paralogs of zinc-dependent proteins could be supplied with cobalt to retain functionality (25). This would also explain the role of cobalt in zinc-starved *S. typhimurium* (38) and the link between Zn and Co homeostasis and Cd resistance in *C. metallidurans*. One of the functions of the CzcCBA transenvelope system, but which is absent in the AE104 strain, could be to prevent import of too much of either metal into the plasmid-bearing *C. metallidurans* CH34 cells. Because ZupT is required for the Zn-Co-Cd connection, this would explain why the CzcCBA efflux complex cannot exist in the absence of ZupT (43).

### Binding of metals to the CobWs

The homologs of the CobWs are associated with the delivery of Zn(II) to Zn-dependent proteins in bacteria and eukaryotes (22, 23, 54, 55). Metal delivery is aided by GTP- or ZTP-hydrolysis (27) in the case of the CobW-ortholog ZagA. CobW1, which is only needed under extreme zinc-starvation conditions in *C. metallidurans* (20), is related to ZagA, ZNG1 from *Saccharomyces cerevisiae,* and CobW from *Rhodobacter capsulatus* (Fig. S7). All these proteins display the typical Walker A-, B-, switch-, and G-binding motifs of GTPases. The only difference between the GTPases and the ZTPase ZagA could be the Cys residue upstream of the highly conserved Asp residue in the G-binding motif (Fig. S1 and S7). With the exception of CobW3, all of these proteins have an internal metal-binding site between the Walker B and the switch motifs, which is highly conserved (GCI/mCC), strongly selective for soft and borderline transition metal cations, and is probably involved in triggering the GTPase activity (56). More importantly, binding of MgGTP pre-conditions this site for acceptance of the correct metal ions (41). Always in dependence of the extant cytoplasmic metal ion concentration, this, nevertheless, assures the correct metalation of a CobW homolog (40), so that CobW_Rcap from *R. capsulatus* accepts Co(II) but YeiR and YjiA accept Zn(II) (41), delivering it to the internal metal-binding site. This metal preference, modeled under *in vivo* conditions, however, was mirrored to some degree by the relationship between the respective proteins (Fig. S7). CobW_Rcap and YeiR form one deeply branched relationship; ZagA, CobW1, and CobW3 a second; YjiA, CobW2, and ZNG1 the third, which is again deeply branched.

In addition to the missing internal metal binding motif in CobW3, this protein and CobW2 contain large His-rich regions, which AlphaFold2 predicts to form a random coil (Fig. S1 and S7). This region is located at the C-terminus of CobW3 but is internal in CobW2. These motifs could indeed be intrinsically disordered protein (IDP) regions, which organize themselves during binding of metal cations. In this scenario, binding of one metal cation to CobW2 would be to the internal metal-binding site and controlled by the *in vivo* concentration of a metal cation among those of competing metal cations. With increasing Co and decreasing Zn concentrations, the probability of Co-binding over Zn-binding would consequently increase.

Binding of metal cations to the putative IDP regions would not be controlled directly by MgGTP (41) but by the proportion of a metal among the other cations present in the cell. CobW3 binds about 8 (6.5 to 9) Zn(II) ions with decreasing affinity when only Zn(II) is present. When incubated with a metal mix, 4 Zn(II), 2 to 3 Ni(II), 1 Co(II), and 1 Cd(II) are bound (20). CobW2, with its large internal His-rich region, is present in two different conformations, binding 0.5 Zn(II) or about 7 Zn(II), respectively, when only zinc is present. When offered a mixture of metal cations, CobW2 precipitates (20). This led to the assumption that CobW2 is a zinc-storage protein that binds up to 7 Zn(II) ions in its “open” conformation and one or none Zn ions in its “closed” conformation. CobW2 could therefore act as a zinc buffer but, as shown by the CobW2-dependent decrease of Co resistance of the ∆*zupT* ∆*cobW3* mutant (Table 6), may also interact in some way with DmeF. With about 2,000 copies per cell and 7 Zn(II) ions bound per protein, CobW2 would be able to store 14,000 Zn(II) of the total 70,000 Zn(II) ions in the cell (17, 20), representing 20% of the cellular zinc. CobW3, on the other hand, has a clear influence on metal import (20), and this is further substantiated by the findings of the current study. CobW3 may be capable of determining the relative proportions of metal cations in the metal mix through differential affinities by binding them at its C-terminal His-rich region. This would consequently affect metal transport depending on the actual metal cations bound.

### Zinc homeostasis in *C*. *metallidurans*

As far as is known, zinc is an essential element for all organisms (23, 57). The divalent Zn(II) transition metal cation has a completely filled 3d^10^ orbital, which prevents the formation of stable octahedral complexes because no empty 3d orbitals are available to accept free electron pairs from metal ligands (58). Thus, Zn(II) forms tetrahedral complexes. These complexes have the function of stabilizing the conformation of proteins such as in the periplasmic Cu-Zn-dependent superoxide dismutase or in the RpoC (beta-prime) subunit of the RNA polymerase (59, 60). Alternatively, Zn(II) can also act as Lewis acid catalyzing biochemical reactions such as in alcohol dehydrogenase, carbonic anhydrase, or FolE_IA-type GTP-cyclohydrolase I, which initiates biosynthesis of the essential cofactor tetrahydrofolate (25, 61).

As *C. metallidurans* typically contains 70,000 to 80,000 Zn(II) per cell when cultivated in standard TMM (8, 17, 43), high external zinc concentrations cause transient accumulation of zinc resulting in the cation being exported by the P_IB2_-type ATPase ZntA in the plasmid-free strain AE104, whereas in the CH34 wild-type strain additional plasmid-encoded zinc efflux pumps eject excess zinc ions. Strain AE104 contains a much higher number of zinc-binding proteins, about 110,000 per cell (17), so that after growth in standard TMM, not all zinc-binding sites are occupied. Half of the zinc-binding proteins are involved in genetic information-processing, with two-thirds of these being zinc-binding ribosomal proteins and the remaining one third being zinc-binding proteins of the RNA polymerase. However, only about 4,709 ± 128 copies of RpoC per cell (17) need a zinc ion for correct folding, which is “checked” by the omega subunit RpoZ before final assembly of the RNAP. The lowest cellular zinc content measured in this study was 7,100 ± 1,200 Zn ions per cell (Table 1) in low zinc medium, which would leave about 2,400 Zn for other essential zinc-dependent proteins, for instance, the periplasmic SodC, with 164 ± 43 copies per cell (17).

An operon in *C. metallidurans* contains two Zur-binding sites at the promoter, is only expressed under extreme zinc starvation, and includes a gene encoding the third COG0523-family protein CobW1. The operon also has genes encoding a metal-promiscuous GTP-cyclohydrolase FolE_IB2 that needs Fe, Mn, or Co for activity, and paralogs of the zinc-dependent proteins CysS, QueD, AllB, as well as a carbonic anhydrase (19, 20). The number of the respective paralogs total about 3,700 proteins (17), so that the 7,100 Zn ions per cell might indeed signify the lowest possible zinc content of these cells. As in case of the zinc-dependent GTP cyclohydrolase FolE_IA and its metal-promiscuous substitutes FolE_IB1 and FolE_IB2 (28, 61, 62), other metal cations might act as Lewis acids in an essential biochemical catalysis, substituting for the lack of zinc.

As a consequence of the Debye-Hückel rule (63) and other constraints, Mg(II) and the divalent transition metal cations should form solvent-shared ions-pairs with the Lewis bases inside the cell and form contact ion-pairs whenever a sufficient number and position of Lewis base ligands are available (53). In contrast, the alkali metal cations Na(I) and K(I) should be mostly fully solvated ions, which counteract the negative charges of the proteins and nucleic acids, but otherwise can enter into solvent-shared ions-pairs with these compounds. The proteome of *C. metallidurans* may have a capacity of nearly 6 million binding sites for divalent metal cations, including those in the 110,000 proteins of the zinc repository (17, 53), which might interact with half of the 10 million Mg(II) ions per cell, if Mg(II) is not out-competed by transition metal cations. Moving from protein to protein, Zn(II) might follow the amino acids on their path to the translating ribosome. The ribosomal proteins and the RNA polymerase contain zinc buffers, with RNAP having additional zinc-binding sites besides the essential one in RpoC (17). In this way, Zn(II) is available to be inserted into nascent proteins during translation, which explains the zinc-dependence of translation (64). Proteins such as ZagA might be required for those proteins that did not, or could not, obtain their zinc during translation, or which have lost it.

Should this hypothetical zinc allocation pipeline become limiting for the cation, other metal cations may outcompete the Mg(II) and follow the amino acids to the ribosome. Cd(II), which belongs to the same group of the periodic system as Zn(II) and also has a completely filled d-orbital, may bind to zinc-binding sites in the absence of competing zinc ions, which explains the observed greater cadmium sensitivity of zinc-starved cells. Should Fe(II) enter this allocation pipeline, zinc-dependent proteins would receive the highly redox-active iron. This situation should be prevented by iron-storage proteins or by its rapid sequestration by Fe-dependent proteins, for example, in the form of Fe-S-clusters (30, 65). *C. metallidurans* does not use Mn(II) and handles Cu(I) by a sophisticated periplasmic copper homeostasis and export from the cytoplasm (8, 66). This leaves Co(II) and Ni(II) as the only other possible metal cations that may follow the zinc allocation pathway.

### Cobalt and nickel

Ni(II) ions have eight electrons in their 3d orbital (58). An octahedral complex would have six electrons in the non-binding and the remaining two electrons in an anti-bonding 3d orbital, so that four ligands are firmly bound and the axial two are only loosely bound. Such Ni(II) complexes appear as square-planar complexes (41). The CnrCBA transenvelope efflux complex is responsible for nickel resistance in *C. metallidurans* (67). It is regulated by the extracytoplasmic sigma factor CnrH, bound in the absence of nickel by the membrane-bound CnrYX complex with CnrX being the sensor for periplasmic Ni(II) (68–70). CnrX binds Ni(II) in a quasi-octahedral complex (71–74). Two adjacent corners of the Ni(II) complex are occupied by the terminal carboxyl group of a glutamate residue. One anti-bonding d-orbital of the central Ni(II) ion contains the two electrons as an electron pair. This allows Ni(II) to accept one more electron pair from the deprotonated carboxyl group of the Glu ligand. The required pairing energy of the electrons is compensated by the release of energy stemming from the mesomeric overlay of one oxygen donating an electron pair to the Ni(II) as fifth ligand and the other double-bonded oxygen is not bonded by Ni(II) (74). This discriminates strongly against Zn(II) but not Co(II); however, *cnr* is only mildly upregulated by Co(II) compared with Ni(II) (70). In *C. metallidurans* CH34 wild type, the CzcCBA efflux pump keeps the periplasmic Co(II) level low, even in medium with a low metal content (75), so that CnrCBA is only produced at high (low mM) nickel, or even higher cobalt concentrations (5, 76).

In comparison to Ni(II), Co(II) has only seven electrons in the 3d orbital, six in the non-bonding d orbitals and one in an anti-bonding d-orbital. Octahedral complexes are possible, with a switch between the formal Co(II) and Co(III) oxidation states allowing the binding of the 6th beta-ligand or weakening of this bond, as for instance in cobalamin during mutase reactions (77). As outlined elsewhere (7), this provides low-spin Co(III) complexes with a low energetic state due to the half-filled 3d orbitals so that Co(III) complexes are kinetically stable, which traps cobalt in cobalamin complexes so that most of the cell-bound cobalt may reside in these complexes. Derivatives of cobalamin are even used to exchange cobalt between cells, allowing them to keep the concentration of unbound Co(II) in the cytoplasm very low (7).

Trapping of cobalt in cobalamin complexes may lower the concentration of Co(II) available for metalation of CobW3 and other proteins, so that Zn(II) may out-compete cobalt here (41). Indeed, *Pseudomonas denitrificans* strains can be used to produce up to 200 mg/L B_12_ under biotechnological conditions (78). This would calculate to about 150 nM of B_12_ in the growth medium, which would need the same Co(II) concentration to allow biosynthesis. *C. metallidurans* contains the genes for cobalamin biosynthesis or uptake; the respective proteins are there, but none of the genes was up- or down-regulated in *C. metallidurans* strain AE104 under metal stress or starvation conditions (79, 80). Although the abundance of the proteins involved in cobalamin biosynthesis seem not to be regulated under conditions of changing metal availability, product removal by trapping may increase the ratio of cytoplasmic Co bound to cobalamin. On the other hand, none of the tested strains exhausted the cobalt content of the growth medium. That would be expected if a strong cobalamin biosynthesis rate traps all the available cobalt in the center of B_12_. At this stage, it cannot be concluded how much of the cytoplasmic cobalt is trapped as B_12_ and how this trapping influences binding of Co(II) to CobW3 or the FolE_IBs (41).

CobW3 bound Zn(II), Ni(II), Co(II), and Cd(II), and each metal should form different complexes by binding to the variety of amino acid residues in the large C-terminal His-rich loop of the protein (Fig. S1). This may allow CobW3 to form a variety of conformations of its C-terminal domain, resulting in different actions of CobW3, for instance, during protein-protein interactions. Although Cd(II) should be exported by the P_IB2_-type ATPases ZntA and CadA (15), *C. metallidurans* cells contain Ni-hydrogenases and maturation proteins like HypB, another member of the COG0523 protein family like the CobWs, even under heterotrophic growth conditions (5), and these proteins should serve as sinks for Ni(II). Therefore, only Co(II) remains to occupy Zn-binding sites and protect them from being bound by Cd(II). To prevent cobalt toxicity, however, the cellular Co(II) level has to be strictly controlled, which is accomplished by the interaction of CobW3, ZupT, DmeF and CobW2 (Fig. S1), and possibly trapping of Co(II) in cobalamin complexes.

## MATERIALS AND METHODS

### Bacterial strains and growth conditions

Strains used for experiments were derivatives of the plasmid-free derivative AE104 of *C. metallidurans* CH34 (5) and are listed in Table S2. Tris-buffered mineral salts medium (5) containing 2 g sodium gluconate/l (TMM) was used to cultivate these strains aerobically with shaking at 30°C. Modified versions of the standard TMM contained different zinc and magnesium concentrations (Table S1). Solid Tris-buffered media contained 20 g agar/L.

### Dose–response growth curves in 96-well plates

Experiments were conducted in TMM. A pre-culture was incubated at 30°C, 200 rpm up to early stationary phase, then diluted 1:20 into fresh medium and incubated for 24 h at 30°C and 200 rpm. Overnight cultures were used to inoculate parallel cultures with increasing metal concentrations in 96-well plates (Greiner). Cells were cultivated for 25 h at 30°C and 1,300 rpm in a neoLab Shaker DTS-2 (neoLab, Heidelberg, Germany) and the optical density was determined at 600 nm in a TECAN Infinite M Nano reader (Tecan Group Ltd., Männedorf, Switzerland) as indicated. To calculate the IC_50_ values (inhibitory concentration: metal concentration that led to turbidity reduction by half) and the corresponding *b*-value (measure of the slope of the sigmoidal dose–response curve), the data were adapted to the formula OD(*c*) =OD0/{1 + exp((*c* - IC_50_)/*b*)}, which is a simplified version of a Hill-type equation as introduced by Pace and Scholtz (81) as published (82). OD(*c*) is the turbidity at a given metal concentration, OD0 that had no added metal, and *c* is the metal concentration.

### Time-dependent growth curves in 48-well plate

Experiments were conducted in TMM. A pre-culture was incubated at 30°C, 200 rpm up to early stationary phase, then diluted 1:20 into fresh medium and incubated for 24 h at 30°C and 200 rpm. Overnight cultures were diluted 50-fold in fresh medium with or without additions in 48-well plates (TPP). The kinetic loop consisted of 90 cycles and was performed in TECAN Spark microplate reader (TECAN, Switzerland). Shaking duration was 2,000 seconds, in orbital mode with an amplitude of 4 mm and frequency of 150 rpm. Optical density was measured at 600 nm at the end of each cycle.

### Genetic techniques

Standard molecular genetic techniques were used (83, 84). For conjugative gene transfer, overnight cultures of donor strain *E. coli* S17/1 (85) and of the *C. metallidurans* recipient strains grown at 30°C in Tris- buffered medium were mixed (1:1) and plated onto nutrient broth agar. After 2 d, the bacteria were suspended in TMM, diluted, and plated onto selective media as previously described (83). Primer sequences are provided in Table S2.

### Gene deletions

Primer sequences are also provided in Table S2. Plasmid pECD1002, a derivate of plasmid pCM184 (86), was used to construct deletion mutants. These plasmids harbor a kanamycin resistance cassette flanked by *loxP* recognition sites. Plasmid pECD1002 additionally carries alterations of 5 bp at each *loxP*-site. Using these mutant *lox* sequences, multiple gene deletions within the same genome are possible without interferences by secondary recombination events (87, 88). Fragments of 300 bp upstream and downstream of the target gene were amplified by PCR, cloned into vector pGEM T-Easy (Promega), sequenced, and further cloned into plasmid pECD1002. The resulting plasmids were used in a double-crossover recombination in *C. metallidurans* strains to replace the respective target gene by the kanamycin-resistance cassette, which was subsequently also deleted by transient introduction of *cre* expression plasmid pCM157 (86). Cre recombinase is a site-specific recombinase from the phage P1 that catalyzes the *in vivo* excision of the kanamycin resistance cassette at the *loxP* recognition sites. The correct deletions of the respective transporter genes were verified by Southern DNA-DNA hybridization. For construction of multiple deletion strains, these steps were repeated. The resulting mutants carried a small open reading frame instead of the wild-type gene to prevent polar effects.

### Inductively-coupled plasma mass spectrometry (ICP-MS)

Cells were incubated in TMM for 20 h at 30°C with shaking at 200 rpm, diluted 20-fold into fresh TMM medium, and shaking was continued at 30°C for 24 h. Cells were diluted 66-fold into fresh medium until 100 Klett was reached (mid-exponential phase of growth). Metals were added, and the cells were left growing until they reached 150 Klett. Ten milliliters of the cells were harvested by centrifugation, washed twice with 50 mM TrisHCl buffer (pH 7.0) containing 10 mM EDTA, and 150 mM NaCl at 4°C. For ICP-MS analysis, HNO3 (trace metal grade; Normatom/PROLABO) was added to the samples to a final concentration of 67% (wt/vol), and the mixture was mineralized at 70°C for 2 h. Samples were diluted to a final concentration of 2% (wt/vol) nitric acid. Indium and germanium were added as internal standards at a final concentration of 1 ppb and 10 ppb each. Elemental analysis was performed via ICP-MS using Cetac ASX-560 sampler (Teledyne, Cetac Technologies, Omaha, Nebraska), a MicroFlow PFA-100 nebulizer (Elemental Scientific, Mainz, Germany), and an ICAP-RQ ICP-MS instrument (Thermo Fisher Scientific, Bremen) operating with a collision cell and flow rates of 4.5 mL × min^−1^ of He/H_2_ [93%/7% (89)], with an Ar carrier flow rate of 0.76 L × min^−1^ and an Ar make-up flow rate at 15 L × min^−1^. An external calibration curve was recorded with ICP-multi-element standard solution XVI (Merck) in 2% (vol/vol) nitric acid. The sample was introduced via a peristaltic pump and analyzed for its metal content. For blank measurement and quality/quantity thresholds, calculations based on DIN32645 TMM were used. The results were calculated from the ppb data as atoms per cell as described (8).

### Pulse-chase experiments with radioactive ^65^Zn

Cells were incubated in TMM for 17 h at 30°C shaking at 200 rpm, diluted 20-fold into a second pre-culture in the medium that was used for the subsequent main culture (TMM, aZn, lZn, and lZn_lMg) and incubated with shaking at 30°C for 24 h. Cells were diluted 50-fold into the main culture, which was incubated with shaking at 30°C at 200 rpm until a turbidity of 150 Klett units was reached (mid-exponential phase of growth). The cells were harvested by centrifugation at 4°C, washed in the same volume of 10 mM TrisHCl (pH 7), suspended in the same volume of 10 mM TrisHCl (pH 7), and kept on ice until needed during the same day. For the experiments, sodium gluconate was added to 6 mL of the cell suspension to a final concentration of 2 g/L directly before the start. At t = 0, radioactive ^65^Zn was added to the cell suspension to a final concentration of 1 µM Zn(II) and 60 nCi/mL. The ^65^ZnCl_2_ was supplied by POLATOM (certificate 022–106722-03622-0001).

The cells were incubated with shaking at 30°C. At 0.25, 5, 10, and 15 min, samples of 500 µL were removed and filtered through a membrane filter (0.2-µm pore size, Whatman cellulose nitrate membrane filters, Cytiva) using a vacuum-driven uptake apparatus. The samples were rapidly washed twice with 5 mL of 50 mM TrisHCl (pH 7) containing 50 mM EDTA. The activity was counted in a Liquid Scintillation Counter (PerkinElmer Tri-Carb 2810 TR) using Ultima Gold (PerkinElmer). The samples were counted twice for 2 min in a window from 0 to 200 keV.

For the chase, non-radioactive zinc was added at t = 20 min to a final concentration of 100 µM. Incubation was continued with shaking at 30°C, and samples were removed at 20.25, 25, 30, 35, and 40 min. They were treated and analyzed as described above for the samples of the uptake period.

A sample of 100 µL was counted to determine the total radioactivity of the ^65^Zn in the cell suspension used for the pulse-chase experiment. From this value, the mol zinc per cpm ratio was derived. For each time sample, the mean value and technical deviation of the two 2 min counts were calculated. Two zero controls were subtracted, one for the background radioactivity at the time of the experiment and one for the chemical adsorption of ^65^Zn by the membrane filter. The resulting value was multiplied with the mol/cpm ration of the respective experiment to give the mol ^65^Zn per 500 µL time sample. The actual cell number in the sample had been determined via an equilibration curve for the turbidity at 600 nm, so that the mol ^65^Zn per cell and subsequently the number of the ^65^Zn atoms per cell could be calculated.

All experiments were performed at least three times. For each individual experiment, the zinc content per cell at 7.5 min was calculated from the 5 min and 10 min values. This value was used to correct the number of atoms per cell for all experiments involving the same mutant and the same growth condition. Experiments with large correction factors were removed and the respective experiment repeated. For each strain and condition, the mean values and deviations of the ^65^Zn atoms per cell were finally calculated. This value was designated as the cellular metal content C(t) for the respective mutant and growth condition.

Pulse-chase with ^65^Zn measured: (i) the initial zinc uptake velocity v_up_(0) at t = 0; (ii) the cellular ^65^Zn content C_20_ at the end of the uptake period (time point represented in Fig. 2 by the horizontal bar); (iii) the extrapolated maximum zinc content after the uptake period C_max_; (iv) the efflux velocity v_eff_ at the beginning of the chase at 20 min; (v) the corresponding initial zinc content C_0_ used to calculate v_eff_; and (vi) and the final zinc content C_40_ at the end of the chase period (Fig. 2, t = 40 min). To obtain these data, the uptake phase up to 20 min of the pulse-chase experiment was adapted to the equation C(t) =C_max_ . t/(K_t_ +t) using the Lineweaver-Burk-like plot 1 /C(t) =1/C_max_ +K_t_/C_max_ . 1 /t. The first deviation by time of the equation C(t) =C_max_ . t/(K_t_ +t) was dC(t)/dt = C_max_ . K_t_ /(K_t_ +t)^2^. At t = 0, this gave the initial uptake rate v_up_(0) = C_max_/K_t_. After the chase after 20 min, the cell-bound zinc content was modeled by the decay function C(t) =C_o_ . e^(-t . t) using the plot ln C(t) =ln C_o_ -t . t. The first deviation by time of the equation C(t) =C_o_ . e^(-t . t) was dC(t)/dt = -t . C_o_ . e^(-t . t). However, at t = 0, this value was the initial net efflux rate v_eff_(0) = -t . C_o_. In contrast to the initial uptake rate that was no net rate because the cells did not contain ^65^Zn at t = 0, v_eff_(0) was a net rate, and the result of the real efflux rate after chase minus the rate of ^65^Zn re-import at this time.

### Experiments with stable ^67^Zn

Stable enriched ^67^Zn was employed to determine (vii) the resident zinc pool (ZP) ZP1 at the beginning of the experiment; (viii) the zinc pools ZP1 and ZP2 after the uptake period; and (ix) finally, these pools ZP1 and ZP2 after the chase period. The cell suspensions were prepared in the respective media as described for the pulse-chase experiments above; however, the respective growth medium was used instead of uptake buffer for these experiments. After a zero sample had been removed for the ICP-MS analysis, isotope-enriched ^67^Zn(II) was added to a final concentration of 1 µM. Incubation was continued with shaking for 20 min, a sample was removed, and the remaining cells were chased with non-enriched Zn(II) added at a final concentration of 100 µM. Incubation was continued for 20 min with shaking at 30°C, and the third sample was removed. The cells in the respective samples were harvested by centrifugation, washed twice with 50 mM TrisHCl buffer (pH 7.0) containing 50 mM EDTA at 0°C, suspended in 50 mM TrisHCl buffer (pH 7.0), and mineralized for the subsequent ICP-MS analysis. The ^67^Zn (94% ^67^Zn) was provided as metal from Nakima Ltd (Savyon, Israel) and oxidized using HCl on ice. The zinc content was verified by ICP-MS.

For the calculation of different zinc pools in the cells, the ratio of ^67^Zn in the isotope-enriched zinc solution (94%) and non-enriched “usual” zinc [4.1% (90)] was used. The ICP-MS measurement calculates the quantity of an element from that of its isotopes, thereby correcting for the % of the natural abundance of the respective isotope. The zinc pool 1 (ZP1) was defined as the cellular zinc pool before addition of isotope-enriched ^67^Zn and was equal to the ^64^Zn ICP-MS result [natural abundance 48.6% (90)]. Similar results were obtained by using ^66^Zn (27.9%) instead of ^65^Zn. Zinc pool 2 (ZP2) was the zinc pool after incubation of the cells with ^67^Zn. ZP2 was the ^67^Zn value coming from the ICP-MS (corrected for a natural abundance 4.1%) minus the ^64^Zn value (0.75% in the ^67^Zn-enriched zinc solution) and the result was divided by 22.2346.

### Statistics

Students’ *t*-test was used, but in most cases, the distance (D) value, D, has been used several times previously for such analyses (10, 91, 92). It is a simple, more useful value than Student’s *t*-test because non-intersecting deviation bars of two values (D > 1) for three repeats always mean a statistically relevant (≥ 95%) difference, provided the deviations are within a similar range. At *n* = 4, significance is ≥97.5%, at *n* = 5 ≥ 99% (significant), and at *n* = 8 ≥ 99.9% (highly significant).

## SUPPLEMENTAL MATERIAL

Supplemental material jb.00226-24-s0001.pdf Tables S1 to S5; Figures S1 to S7.

ASM does not own the copyrights to Supplemental Material that may be linked to, or accessed through, an article. The authors have granted ASM a non-exclusive, world-wide license to publish the Supplemental Material files. Please contact the corresponding author directly for reuse.
