# Supplementary material for: The metal-binding GTPases CobW2 and CobW3 are at the crossroads of zinc and cobalt homeostasis in Cupriavidus metallidurans
Source: J Bacteriol. 2024 Jul 23;206(8):e00226-24. doi: 10.1128/jb.00226-24 (PMC11340326; doi:10.1128/jb.00226-24)
Supplement: Supplemental material — Tables S1 to S5; Figures S1 to S7. [file jb.00226-24-s0001.pdf]

Supplementary Tables and Figures

Supplementary Table S1. Zn and Co content of the Tris Minimal Medium[a]

TMM derivative	Zinc source	nM Zn(II)	nM Co(II)	µM Mg(II)
High zinc (hZn)	SL6, Na_2SO_4	392±110	112±5	1000
Ambient zinc (aZn)	Adjusted with $ZnCl_2$	200	86±17	1000
Standard TMM	SL6, Na_2SO_4 x 10 H_2O[b]	160±36	106±10	1000
Moderate zinc (mZn)	SL6, Na_2SO_4 x 10 H_2O[b]	64±9	86±17	1000
Zinc starvation (lZn)	Na_2SO_4 x 10 H_2O	39±8	1.8±1.4	1000
Metal starvation (lZn_lMg)	Na_2SO_4 x 10 H_2O	39±8	1.8±1.4	100

[a] The metal concentrations were determined by ICP-MS. [b]Different charges.

Supplementary Table S2. Bacterial strains and DNA-primers

Strain	Relevant markers	Reference
	Cupriavidus metallidurans	
AE104	no plasmid	(1)
DN515	AE104 Δ*zupT*	(2)
DN717	AE104 Δ*cobW2::kan*	(3)
DN818	AE104 Δ*cobW3*	(3)
DN718	AE104 Δ*zupT* Δ*cobW2::kan*	(3)
DN824	AE104 Δ*zupT* Δ*cobW3*	(3)
DN586	AE104 Δ*dmeF*	(4)
DNA38	AE104 Δ*dmeF* ΔcobW2::kan	This work
DNA39	AE104 Δ*dmeF* ΔcobW3	This work
DNA40	AE104 Δ*dmeF* ΔcobW3 ΔcobW2::kan	This work
DNA41	AE104 Δ*dmeF* Δ*zupT*	This work
DNA42	AE104 Δ*dmeF* Δ*zupT* ΔcobW2::kan	This work
DNA43	AE104 Δ*dmeF* Δ*zupT* ΔcobW3	This work
DNA44	AE104 Δ*dmeF* Δ*zupT* ΔcobW3 ΔcobW2::kan	This work
DNA34	AE104 ΔRmet_0197	This work
DNA35	AE104 ΔRmet_0199	This work
DNA36	AE104 ΔRmet_0200	This work

DNA-Primers

Name/orientation	5'->3- sequence	Position
Gene disruptions		
Rmet_0127 PstI Dis →	AAACTGCAGGCTCGACAAGCAGGAAGAAG	binds 412 bp downstream of ATG_{Rmet_0127}
Rmet_0127 XbaI Dis ←	AAATCTAGAGCAATCGGTGCCGCAGTGT	binds 376 bp upstream of TGA_{Rmet_0127}
Cre-lox		
Cre 0125 Age →	AAAACCGGTTCGAATCCGGCGACTATGGCTG	binds 332 bp upstream of ATG_{cobW3}
Cre 0125 Apa ←	AAAGGGCCCGATGATCGATGTTGATGCAACAAAG	binds directly upstream of ATG_{cobW3}
Cre 0125 NotI →	AAAGCGGCCGCAGTCCTGCACCCTCTCTC	binds directly downstream of TGA_{cobW3}
Cre 0125 NcoI ←	AAACCATGGCCAGGTTGGCTGGCTTGAC	binds 311 bp downstream of TGA_{cobW3}
zupT 1 NcoI →	AAACCATGGCGGTGCTGGGTCTGT	binds 520 bp upstream of ATG_{zupT}
zupT 2 NotI ←	AAAGCGGCCGCGCACCCCGACTGAAAAGCTCA	binds directly downstream of ATG_{zupT}
zupT 3 AgeI →	AAAACCGGTTCGAACTGATGTCGCTGG CGG	binds directly upstream of TGA_{zupT}
zupT 4 SacI ←	AAAGAGCTCGTGAAGACCGTGGATGCCGAC	binds 425bp downstream of TGA_{zupT}

1485 delta dmeF AgeI →	AGGACCGGTCGTCGGCATCCTGGTGGGT	binds 638bp upstream of ATG_{dmeF}
1898 delta dmeF MluI ←	AAGACGCGTCGCGTGCCGAGAGCTTGTCAC	binds 204bp upstream of ATG_{dmeF}
3132 delta dmeF NotI →	TCAGCGGCCGCGCGACGATGGCCGAGACG	binds 56bp upstream of TAG_{dmeF}
3512 delta dmeF BglII ←	GGGAGATCTCACCTTCCTCGGCTCGCTCG	binds 341 downstream of TAG_{dmeF}
Promo_0197Mun 1047up →	AAACAATTGCCGCAATCGGAGCGGTAC	binds 351 bp upstream of ATG_{Rmet_0197}
Promo_0197Not1 368down ←	AAAGCGGCCGCCCTTGCTTTCGAGAATTTCCG	Binds 26 bp upstream of ATG_{Rmet_0197}
Promo_0197Apa 1735up →	AAAGGGCCCGTCGAGGCGATTGGCGAT	binds 224 bp downstream of ATG_{Rmet_0197}
Promo_0197Age 2083down ←	AAAACCGGTGATCGCGACGTCGCATCC	binds 407 bp upstream of TGA_{Rmet_0197}
_0199 Mun 672 up →	AAACAATTGGATGTGATCGACCACGGC	binds 311 bp upstream of ATG_{Rmet_0199}
_0199 Apa 1898 up →	AAAGGGCCCGACCCCGCCGCGCCATAT	binds 12 bp upstream of ATG_{Rmet_0199}
_0199 Age 2236 down ←	AAAACCGGTTGGCCAGTGCGTGCGAGC	Binds 178 bp downstream of ATG_{dmeF}
_0199 Not 1013 down ←	AAAGCGGCCGCGAAGAACGCGAGATCGGA	binds 12 bp downstream of ATG_{Rmet_0199}
_0200-01 Mun 868 up →	AAACAATTGCCGGCAAGACTGCCGCGC	binds 77 bp upstream of ATG_{Rmet_0199}
_0200-01 Not 1192 down ←	AAAGCGGCCGCCCCTGCTCGGCGAGCCCG	binds 128 bp downstream of ATG_{Rmet_0200}
_0200-01 Apa 3099 up →	AAAGGGCCCATGGCACGGCTCGGCCGC	binds 607 bp downstream of TGA_{Rmet_0200}
_0200-01 Age 3443 down ←	AAAACCGGTCGCCGCGGTCGTGAGCGA	binds 840 bp downstream of TGA_{Rmet_0200}

Supplementary Table S3. Metal content of *C. metallidurans* strain AE104 and its ∆*zupT* mutant under various growth conditions

Strain	Medium		Metal atoms per cell								
	Zn	Co	Mg	Ca	Mn	Fe	Co	Ni	Cu	Zn	Mo
	nM	nM	10^6	10^3		10^3	10^3	10^3	10^3	10^3	10^3
	High zinc										
AE104	400	110	11.4±1.0	112±79	252±292	692±68	3.7±1.1	1.90±1.56	9.07±3.99	75.8±7.7	1.91±1.18
Δ*zupT*	400	110	10.5±0.8	133±25	142±90	710±91	6.4±0.9	3.59±0.80	8.01±1.49	42.8±4.3	2.58±0.26
	Standard TMM										
AE104	160	110	12.1±1.3	140±56	353±370	733±87	21.3±10.7	2.99±1.09	7.70±2.10	68.4±12.4	3.05±0.39
Δ*zupT*	160	110	10.7±0.6	164±59	202±59	765±52	7.3±1.9	4.28±0.84	8.37±3.02	28.6±4.8	2.56±0.23
	Low zinc ± 150 nM Zn(II)										
AE104	39	2	11.4±1.3	114±30	218±61	720±84	0.2±0.0	2.52±0.64	2.97±1.24	27.5±11.0	2.56±0.35
AE104	39	100	11.6±1.2	163±19	104±54	689±32	40.2±3.6	1.89±0.43	4.34±1.50	18.1±9.6	2.69±0.17
AE104	39	150	9.8±0.6	117±38	195±99	618±46	40.0±7.4	1.86±0.63	3.88±0.84	19.7±5.8	2.49±0.43
AE104	39	300	10.0±0.9	139±52	56±44	618±71	57.2±16.5	0.58±0.17	4.14±1.61	21.6±8.7	2.41±0.49
AE104	190	100	13.2±2.1	256±47	112±25	772±168	25.6±3.6	2.67±1.67	3.19±0.86	83.6±6.1	2.48±0.11
AE104	190	150	10.5±0.6	145±25	141±47	606±46	20.6±3.5	0.34±0.05	3.19±1.18	65.4±4.7	1.88±0.33
AE104	190	300	12.1±1.4	148±29	53±29	649±51	24.9±3.2	0.79±0.21	4.00±1.41	74.5±8.2	2.14±0.27
Δ*zupT*	39	2	10.8±0.1	107±24	282±22	730±29	0.1±0.1	5.20±0.75	4.76±1.99	19.9±2.4	2.13±0.16
Δ*zupT*	39	100	10.7±0.4	166±33	125±53	664±33	35.2±3.3	1.94±0.54	3.65±1.64	14.6±2.2	2.24±0.17
Δ*zupT*	39	150	10.2±0.9	157±38	166±99	694±58	44.8±5.9	7.06±4.69	9.04±5.06	14.5±6.2	2.19±0.24
Δ*zupT*	39	300	10.4±0.9	179±45	131±69	690±45	46.5±19.8	2.18±0.99	6.77±3.64	37.1±8.5	1.97±0.21
Δ*zupT*	190	100	35.4±1.9	82±85	172±112	601±10	5.4±0.5	0.46±0.28	4.18±0.10	32.5±2.8	1.44±0.30
Δ*zupT*	190	150	34.8±4.5	143±18	bdl	604±44	8.2±0.8	0.26±0.19	2.24±0.86	23.3±3.5	1.57±0.32
Δ*zupT*	190	300	38.5±4.2	158±22	123±169	642±52	14.1±4.6	2.12±2.26	3.92±1.40	25.8±4.4	1.77±0.38

Bld, below detection limit.

Supplementary Table S4. Metal content of mutants with ∆*cobW* deletions.

Metal content in AE104	Mg, 10^6	Ca, 10^3	Mn	Fe, 10^3	Co, 10^3	Ni, 10^3	Cu, 10^3	Zn, 10^3	Mo, 10^3
	12.1±1.2	249±235	174±64	701±59	20.0±8.7	2.76±0.81	6.37±1.41	64.7±11.7	2.92±0.38
Strains									
<u>No addition</u>									
AE104	1.00 (0.0)	1.00 (0.0)	1.00 (0.0)	1.00 (0.0)	1.00 (0.0)	1.00 (0.0)	1.00 (0.0)	1.00 (0.0)	1.00 (0.0)
∆cobW3	0.91 (0.5)	0.56 (0.4)	0.94 (0.1)	0.92 (0.6)	**0.19 (1.6)**	1.13 (0.2)	0.98 (0.1)	0.93 (0.3)	0.80 (1.0)
∆cobW2::dis	0.91 (0.5)	0.53 (0.5)	0.60 (0.5)	0.93 (0.5)	0.66 (0.5)	0.89 (0.2)	0.91 (0.3)	0.99 (0.0)	0.83 (1.0)
∆cobW3 ∆cobW2::dis	0.87 (0.8)	0.62 (0.4)	0.92 (0.1)	0.91 (0.6)	**0.18 (1.8)**	1.13 (0.2)	1.00 (0.0)	0.92 (0.3)	0.81 (1.1)
∆zupT	0.88 (0.7)	0.70 (0.3)	0.95 (0.1)	1.04 (0.2)	**0.36 (1.3)**	1.43 (0.5)	1.01 (0.0)	**0.42 (2.3)**	0.84 (0.7)
∆zupT ∆cobW3	1.14 (0.9)	0.79 (0.6)	0.57 (0.5)	0.98 (0.1)	**1.35 (1.0)**	1.50 (0.9)	1.67 (1.0)	1.12 (0.5)	1.01 (0.0)
∆zupT ∆cobW2::dis	1.09 (0.3)	0.98 (0.0)	**0.37 (1.1)**	0.89 (0.5)	0.99 (0.0)	0.75 (0.4)	1.66 (0.5)	1.10 (0.5)	0.97 (0.1)
∆zupT ∆cobW3 ∆cobW2::dis	1.05 (0.4)	1.06 (0.1)	0.48 (0.8)	0.90 (0.7)	1.09 (0.3)	1.34 (0.7)	0.92 (0.3)	1.26 (0.8)	1.01 (0.1)
∆dmeF	**2.39 (3.8)**	0.60 (0.3)	1.61 (0.5)	0.85 (0.8)	1.04 (0.0)	0.92 (0.2)	0.97 (0.1)	0.92 (0.3)	0.77 (0.8)
∆dmeF ∆cobW3	0.96 (0.2)	1.09 (0.1)	0.97 (0.0)	0.91 (0.5)	**0.24 (2.2)**	1.95 (0.8)	0.90 (0.2)	0.99 (0.0)	0.82 (0.7)
∆dmeF ∆cobW2::dis	1.18 (0.5)	1.62 (0.6)	0.98 (0.0)	0.94 (0.3)	0.71 (0.6)	1.13 (0.3)	0.97 (0.1)	1.16 (0.4)	1.01 (0.0)
∆dmeF ∆cobW3 ∆cobW2::dis	0.97 (0.2)	1.04 (0.1)	0.37 (0.9)	0.95 (0.3)	**0.29 (1.9)**	1.43 (0.8)	0.90 (0.2)	0.96 (0.2)	0.84 (0.6)
∆fieF	0.94 (0.3)	0.38 (0.6)	0.60 (0.7)	0.91 (0.5)	0.97 (0.0)	0.90 (0.2)	0.88 (0.4)	0.94 (0.2)	0.91 (0.4)
∆fieF ∆cobW3	0.96 (0.5)	**1.53 (1.4)**	1.45 (0.5)	0.87 (0.9)	**0.46 (1.2)**	**2.72 (5.8)**	**1.22 (1.1)**	1.09 (0.4)	0.85 (0.9)
∆fieF ∆cobW2::dis	1.05 (0.3)	**2.00 (2.4)**	0.84 (0.3)	0.86 (0.9)	0.75 (0.4)	**2.88 (2.8)**	1.00 (0.0)	1.02 (0.1)	0.90 (0.5)
∆dmeF ∆fieF	0.95 (0.4)	0.27 (0.7)	1.38 (0.4)	1.10 (0.6)	1.39 (0.7)	1.30 (0.7)	1.14 (0.4)	0.89 (0.5)	1.10 (0.5)
∆dmeF ∆zupT	2.11 (2.5)	1.16 (0.1)	**3.65 (1.9)**	1.08 (0.4)	0.82 (0.3)	0.93 (0.2)	0.95 (0.2)	0.55 (2.0)	1.07 (0.2)
∆dmeF ∆zupT ∆cobW3	1.08 (0.3)	0.56 (0.6)	0.68 (0.7)	0.90 (0.6)	1.10 (0.6)	0.79 (0.9)	1.17 (0.8)	1.01 (0.1)	0.85 (0.5)
∆dmeF ∆zupT cobW2::dis	1.00 (0.0)	0.46 (0.8)	0.65 (0.7)	0.78 (1.6)	0.82 (0.9)	0.88 (0.8)	0.80 (2.5)	0.86 (0.5)	0.84 (0.5)
∆dmeF∆zupT∆cobW3 cobW2d	0.96 (0.2)	0.71 (0.3)	0.63 (0.5)	0.77 (1.0)	0.78 (0.8)	0.84 (0.4)	1.56 (0.7)	1.01 (0.1)	0.78 (0.7)
<u>plus 1 µM Co(II)</u>									
AE104	1.08 (0.5)	0.35 (0.6)	0.76 (0.4)	1.11 (0.6)	1.20 (0.4)	1.48 (0.6)	1.56 (0.7)	1.16 (0.6)	1.48 (0.5)
∆cobW3	1.12 (0.8)	**0.50 (2.0)**	1.42 (0.3)	1.10 (0.9)	**3.84 (3.1)**	**2.77 (2.3)**	**1.78 (2.8)**	1.17 (0.9)	1.14 (1.0)
∆cobW2::dis	1.10 (0.7)	**0.59 (1.7)**	2.93 (1.0)	1.05 (0.4)	**1.81 (1.1)**	**2.95 (2.0)**	**1.82 (2.3)**	1.08 (0.6)	1.19 (2.0)
∆cobW3 ∆cobW2::dis	1.11 (0.9)	0.62 (0.7)	2.05 (0.9)	1.03 (0.3)	**4.50 (4.0)**	**2.26 (4.3)**	**2.02 (2.2)**	1.08 (0.5)	1.20 (1.3)
∆zupT	1.07 (0.5)	**0.54 (1.1)**	**3.08 (1.5)**	1.01 (0.0)	**3.42 (5.3)**	1.40 (0.6)	**1.78 (2.0)**	1.29 (1.1)	1.13 (0.8)
∆zupT ∆cobW3	0.89 (0.9)	**0.60 (1.3)**	2.35 (0.7)	1.01 (0.1)	**3.04 (5.3)**	0.81 (0.6)	0.96 (0.1)	1.08 (1.0)	1.28 (2.6)
∆zupT ∆cobW2::dis	0.87 (0.6)	**0.47 (1.4)**	**5.46 (1.7)**	0.99 (0.1)	**3.54 (7.6)**	1.36 (0.8)	0.80 (0.2)	1.04 (0.4)	1.17 (0.8)
∆zupT ∆cobW3 ∆cobW2::dis	0.91 (1.1)	**0.53 (1.2)**	2.09 (0.9)	0.98 (0.2)	**3.11 (5.2)**	1.00 (0.0)	1.44 (1.0)	0.94 (0.3)	1.30 (2.5)
∆dmeF	0.98 (0.1)	1.26 (0.3)	1.49 (0.4)	0.95 (0.3)	**3.47 (3.8)**	**3.11 (1.5)**	1.68 (1.0)	1.03 (0.1)	1.05 (0.2)
∆dmeF ∆cobW3	1.03 (0.3)	0.64 (0.5)	1.65 (0.8)	1.05 (0.3)	**8.29 (21)**	**2.49 (2.1)**	**1.67 (1.2)**	1.04 (0.3)	1.36 (1.1)
∆dmeF ∆cobW2::dis	0.78 (0.9)	**0.25 (1.7)**	0.67 (0.3)	0.91 (0.5)	**4.24 (6.8)**	**3.54 (1.3)**	**1.67 (1.3)**	0.84 (0.5)	1.02 (0.1)
∆dmeF ∆cobW3 ∆cobW2::dis	1.04 (0.2)	0.71 (0.7)	**4.89 (1.4)**	0.97 (0.2)	**7.58 (14)**	**3.29 (2.3)**	1.48 (0.9)	0.98 (0.1)	1.17 (1.1)

∆fieF	1.12 (0.9)	0.81 (0.5)	0	1.09 (0.5)	1.21 (0.4)	**3.04 (2.2)**	**1.86 (2.0)**	1.23 (1.1)	1.02 (0.1)
∆fieF ∆cobW3	1.09 (1.1)	**0.35 (3.7)**	0.72 (0.4)	1.24 (2.1)	**2.48 (2.9)**	0.92 (0.2)	1.16 (0.8)	1.03 (0.2)	1.17 (1.3)
∆fieF ∆cobW2::dis	0.98 (0.1)	**0.58 (1.4)**	3.94 (0.9)	1.27 (1.2)	**1.71 (1.2)**	0.78 (0.8)	**1.70 (1.2)**	1.06 (0.3)	1.12 (0.8)
∆dmeF ∆zupT	0.99 (0.0)	**0.28 (1.2)**	**0.33 (1.6)**	0.82 (0.8)	**3.40 (5.7)**	**4.79 (2.3)**	1.00 (0.0)	0.93 (0.4)	0.86 (0.4)
∆dmeF ∆zupT ∆cobW3	0.84 (0.7)	0.52 (0.6)	1.07 (0.1)	0.78 (1.2)	**3.07 (8.5)**	**6.20 (7.9)**	0.81 (0.6)	1.08 (0.3)	0.92 (0.4)
∆dmeF ∆zupT cobW2::dis	0.87 (1.3)	0.71 (0.4)	0.49 (0.8)	0.89 (1.0)	**4.10 (4.1)**	**4.83 (7.2)**	**1.39 (4.9)**	1.18 (0.5)	0.93 (0.4)
∆dmeF∆zupT∆cobW3 cobW2d	1.19 (1.2)	0.62 (0.4)	0.68 (0.4)	1.06 (0.2)	**6.36 (6.7)**	**6.42 (6.7)**	0.70 (0.5)	1.18 (0.9)	1.28 (1.1)
plus 2.5 µM Co(II)									
AE104	**1.28 (1.1)**	0.45 (0.5)	0.87 (0.3)	0.96 (0.2)	**1.82 (1.1)**	**3.69 (3.5)**	1.10 (0.3)	0.86 (0.4)	1.08 (0.3)
∆cobW3	0.91 (0.3)	0.82 (0.6)	0.48 (0.6)	0.96 (0.4)	**3.57 (2.4)**	**2.75 (1.2)**	**1.69 (1.3)**	0.96 (0.3)	1.10 (0.8)
∆cobW2::dis	0.81 (0.8)	0.70 (1.4)	**0.00 (1.3)**	0.92 (0.7)	1.27 (0.8)	**2.40 (1.2)**	1.61 (1.0)	0.89 (0.9)	1.09 (1.7)
∆cobW3 ∆cobW2::dis	1.10 (0.6)	0.68 (1.3)	0.81 (0.3)	0.99 (0.1)	**4.21 (4.8)**	**2.78 (1.9)**	1.19 (0.9)	0.98 (0.1)	1.14 (1.4)
∆dmeF	0.96 (0.2)	1.09 (0.1)	0.70 (0.3)	0.89 (0.8)	**2.64 (2.1)**	**3.57 (4.2)**	1.35 (0.8)	0.91 (0.6)	0.97 (0.1)
∆dmeF ∆cobW3	0.93 (0.3)	0.79 (0.7)	0.62 (0.6)	0.94 (0.4)	**5.60 (5.1)**	1.86 (1.0)	1.46 (1.1)	0.85 (1.1)	1.15 (1.2)
∆dmeF ∆cobW2::dis	0.77 (0.9)	**0.50 (1.1)**	0.49 (0.4)	0.92 (0.6)	**3.04 (3.3)**	**3.32 (3.7)**	1.55 (0.7)	0.80 (1.1)	1.00 (0.0)
∆dmeF ∆cobW3 ∆cobW2::dis	1.02 (0.1)	**0.57 (1.2)**	16.4 (0.8)	1.04 (0.2)	**5.07 (4.9)**	6.95 (1.0)	**1.69 (1.6)**	0.97 (0.1)	7.27 (0.8)
plus 5 µM Co(II)									
AE104	0.96 (0.2)	0.42 (0.6)	0.95 (0.1)	0.94 (0.4)	**2.73 (2.0)**	0.93 (0.1)	1.11 (0.2)	0.96 (0.1)	1.07 (0.3)
∆cobW3	1.02 (0.2)	0.80 (0.5)	0.61 (0.4)	0.94 (0.8)	**11.9 (7.5)**	0.72 (0.7)	0.79 (0.9)	0.87 (0.8)	1.13 (1.4)
∆cobW2::dis	0.92 (0.7)	0.95 (0.1)	0.72 (0.3)	0.86 (1.7)	**3.19 (4.2)**	0.58 (0.9)	0.67 (2.4)	0.86 (0.6)	1.03 (0.3)
∆cobW3 ∆cobW2::dis	1.01 (0.1)	0.64 (1.0)	1.18 (0.2)	0.89 (1.0)	**10.9 (8.4)**	**0.51 (1.6)**	0.84 (0.4)	0.91 (0.7)	1.09 (0.9)
∆zupT	1.05 (0.7)	0.79 (0.5)	0.85 (0.1)	0.86 (1.2)	**8.70 (19.0)**	**0.46 (1.4)**	0.67 (1.3)	1.09 (0.3)	1.04 (0.3)
∆zupT ∆cobW3	0.87 (1.7)	0.94 (0.2)	1.82 (0.8)	0.88 (1.2)	**6.36 (7.6)**	**0.62 (1.8)**	0.63 (0.9)	1.06 (0.4)	1.11 (1.7)
∆zupT ∆cobW2::dis	0.92 (0.4)	0.86 (0.5)	3.66 (1.0)	0.90 (0.6)	**7.43 (9.4)**	**0.46 (1.6)**	0.63 (0.4)	1.06 (0.8)	1.08 (0.4)
∆zupT ∆cobW3 ∆cobW2::dis	0.95 (0.6)	0.73 (0.6)	1.22 (0.3)	0.90 (1.4)	**7.67 (11.4)**	**0.37 (5.2)**	0.95 (0.5)	0.86 (0.9)	1.25 (3.0)
∆dmeF	0.84 (0.9)	0.87 (0.3)	0.72 (0.5)	0.77 (1.4)	**6.24 (5.4)**	0.98 (0.1)	0.79 (0.5)	0.67 (1.8)	0.86 (0.6)
∆dmeF ∆cobW3	0.81 (1.2)	0.76 (0.6)	1.50 (1.0)	0.77 (1.6)	**23.4 (10.0)**	0.56 (0.8)	0.80 (0.6)	**0.63 (2.4)**	0.92 (0.9)
∆dmeF ∆cobW2::dis	**0.63 (1.5)**	0.62 (0.8)	0.50 (0.4)	0.70 (1.6)	**10.3 (8.0)**	0.70 (0.7)	1.01 (0.0)	**0.47 (2.5)**	0.93 (0.3)
∆dmeF ∆cobW3 ∆cobW2::dis	0.75 (1.5)	1.48 (0.4)	1.27 (0.3)	0.65 (2.5)	**23.5 (17.3)**	0.72 (0.8)	0.81 (0.6)	**0.59 (2.0)**	0.95 (0.4)

The cells were cultivated in **standard TMM** with the indicated concentrations of added Co(II). The ratios Q with the D values in parenthesizes are indicated. For the cells cultivated without added cobalt, the ∆*zupT,* ∆*dmeF* and ∆*fieF* single and double mutants were compared to the parent AE104 and all the respective ∆*cobW* mutants with their AE104, ∆*zupT,* ∆*dmeF* or ∆*fieF* background. The cells cultivated with added cobalt were compared to those without added cobalt in the first part of the table. Three repeats, bold-faced are all values with (0.66 < Q or Q > 1.5) AND D > 1.

Supplementary Table S5. Other metals in pulse-chase experiments with stable ^{67}Zn [a]

Strain/Condition		10^6 Mg	10^3 Ca	Mn	10^3 Fe	10^3 Co	10^3 Ni	10^3 Cu
AE104 mZn	Atoms per cell	12.5±0.9	258±55	575±306	1007±86	9.73±1.06	5.97±1.77	3.90±0.34
		ratio of the value of strain AE104 in mZn medium						
Ambient Zn		Mg ratio	Ca ratio	Mn ratio	Fe ratio	Co ratio	Ni ratio	Cu ratio
AE104	Initial content	1.00±0.07	1.00±0.21	1.00±0.09	1.00±0.53	1.00±0.11	1.00±0.30	1.00±0.09
	Uptake	1.01±0.02	0.94±0.24	0.98±0.04	0.65±0.40	0.92±0.13	0.86±0.12	0.89±0.04
	Chase	1.01±0.10	0.87±0.19	0.93±0.11	*0.48±0.28*	0.86±0.15	0.67±0.04	1.16±0.08
Δ*zupT*	Initial content	0.79±0.01	0.92±0.02	0.23±0.05	0.71±0.01	***0.53±0.02***	***0.33±0.02***	0.87±0.06
	Uptake	0.78±0.06	0.86±0.06	0.20±0.02	0.71±0.05	***0.52±0.04***	***0.23±0.03***	0.80±0.02
	Chase	0.70±0.01	0.75±0.04	0.10±0.04	*0.64±0.00*	***0.46±0.01***	***0.15±0.01***	0.82±0.13
Δ*cobW2*	Initial content	0.81±0.05	1.00±0.07	0.25±0.02	0.81±0.04	***0.56±0.03***	***0.27±0.02***	0.94±0.10
	Uptake	0.86±0.08	0.94±0.04	0.16±0.03	0.75±0.02	***0.52±0.02***	***0.17±0.02***	0.86±0.06
	Chase	0.78±0.04	0.78±0.08	0.28±0.25	0.69±0.08	***0.46±0.07***	***0.28±0.22***	1.24±0.56
Δ*cobW3*	Initial content	0.86±0.05	1.07±0.07	0.52±0.17	0.81±0.07	***0.31±0.02***	***0.35±0.03***	1.01±0.03
	Uptake	0.87±0.09	0.94±0.04	0.43±0.18	0.74±0.03	***0.30±0.01***	***0.22±0.01***	0.92±0.03
	Chase	0.76±0.07	8.88±10.62	4.45±5.54	0.76±0.06	***0.28±0.01***	***0.15±0.03***	1.07±0.11
Δ*zupT* Δ*cobW2*	Initial content	0.96±0.03	1.12±0.06	0.20±0.03	0.84±0.01	***0.53±0.03***	***0.31±0.02***	0.88±0.07
	Uptake	0.90±0.04	0.99±0.10	0.19±0.02	0.76±0.03	***0.48±0.04***	***0.22±0.01***	0.80±0.05
	Chase	0.85±0.03	0.91±0.04	0.09±0.02	0.72±0.02	***0.45±0.03***	***0.16±0.02***	0.97±0.16
Δ*zupT* Δ*cobW3*	Initial content	0.78±0.06	0.91±0.10	0.49±0.18	0.81±0.04	0.71±0.02	***0.62±0.03***	0.84±0.11
	Uptake	0.87±0.01	0.85±0.09	0.40±0.15	0.75±0.02	***0.67±0.01***	***0.54±0.01***	0.75±0.06
	Chase	0.71±0.08	0.77±0.09	0.28±0.13	0.68±0.04	***0.62±0.02***	***0.48±0.01***	0.74±0.05

Continued

Content ratio		Mg ratio	Ca ratio	Mn ratio	Fe ratio	Co ratio	Ni ratio	Cu ratio
<u>Low zinc medium</u>								
AE104	Initial content	1.05±0.03	0.81±0.09	0.95±0.04	*0.48±0.05*	*0.02±0.00*	0.80±0.07	*0.55±0.08*
	Uptake	1.07±0.01	0.77±0.13	0.92±0.01	1.42±0.74	*0.01±0.00*	0.83±0.03	*0.57±0.07*
	Chase	1.06±0.08	0.66±0.18	0.85±0.07	*0.23±0.04*	*0.01±0.00*	0.81±0.13	*0.71±0.04*
Δ*zupT*	Initial content	0.79±0.03	0.87±0.06	0.29±0.03	0.79±0.04	*0.02±0.00*	*0.21±0.02*	*0.35±0.10*
	Uptake	0.78±0.01	1.98±1.43	0.29±0.07	0.74±0.02	*0.01±0.00*	*0.15±0.01*	*0.32±0.12*
	Chase	0.76±0.03	0.78±0.08	0.22±0.07	0.71±0.03	*0.01±0.00*	*0.17±0.03*	*0.42±0.17*
Δ*cobW2*	Initial content	0.73±0.02	0.73±0.05	0.38±0.03	0.67±0.06	*0.05±0.00*	*0.44±0.05*	*0.40±0.12*
	Uptake	0.72±0.02	*0.64±0.03*	0.30±0.04	*0.62±0.04*	*0.05±0.00*	*0.32±0.01*	*0.33±0.02*
	Chase	0.71±0.01	*0.65±0.06*	0.21±0.02	*0.60±0.02*	*0.05±0.00*	*0.26±0.01*	*0.40±0.08*
Δ*cobW3*	Initial content	0.76±0.03	0.78±0.03	0.43±0.08	0.67±0.04	*0.03±0.00*	*0.39±0.01*	*0.39±0.10*
	Uptake	0.79±0.01	1.02±0.26	1.17±1.10	0.68±0.01	*0.03±0.01*	*0.33±0.05*	*0.57±0.19*
	Chase	0.78±0.05	0.69±0.01	0.20±0.03	*0.66±0.06*	*0.02±0.00*	*0.25±0.01*	*0.42±0.12*
Δ*zupT* Δ*cobW2*	Initial content	0.72±0.02	0.78±0.03	0.46±0.05	0.69±0.01	*0.05±0.00*	*0.38±0.01*	*0.50±0.15*
	Uptake	0.74±0.02	0.72±0.05	0.36±0.03	0.67±0.01	*0.05±0.00*	*0.26±0.01*	*0.42±0.17*
	Chase	0.70±0.01	*0.60±0.05*	0.31±0.04	*0.63±0.02*	*0.04±0.00*	*0.23±0.01*	*0.55±0.15*
Δ*zupT* Δ*cobW3*	Initial content	0.67±0.03	0.75±0.12	0.39±0.09	*0.64±0.02*	*0.02±0.01*	*0.54±0.04*	*0.38±0.05*
	Uptake	*0.65±0.02*	0.69±0.08	0.34±0.09	*0.63±0.04*	*0.02±0.01*	*0.48±0.03*	*0.35±0.04*
	Chase	*0.65±0.02*	*0.66±0.09*	0.30±0.09	*0.63±0.03*	*0.02±0.01*	*0.47±0.03*	*0.47±0.10*

Continued

Content ratio		Mg ratio	Ca ratio	Mn ratio	Fe ratio	Co ratio	Ni ratio	Cu ratio
Low Zn, low Mg								
AE104	Initial content	1.03±0.02	0.91±0.25	0.95±0.01	*0.56±0.05*	*0.01±0.00*	1.14±0.10	*0.26±0.04*
	Uptake	1.06±0.01	0.83±0.11	0.90±0.02	*0.45±0.05*	*0.01±0.00*	1.03±0.08	*0.24±0.03*
	Chase	1.05±0.07	0.79±0.26	0.88±0.06	*0.34±0.04*	*0.01±0.00*	0.98±0.04	*0.47±0.13*
Δ*zupT*	Initial content	0.78±0.01	0.95±0.03	0.61±0.02	0.74±0.03	*0.01±0.00*	*0.46±0.01*	*0.32±0.12*
	Uptake	0.77±0.03	0.79±0.04	0.46±0.06	0.68±0.03	*0.01±0.00*	*0.29±0.01*	*0.27±0.09*
	Chase	0.72±0.04	0.73±0.07	0.33±0.04	*0.62±0.04*	*0.01±0.00*	*0.25±0.02*	*0.31±0.07*
Δ*cobW2*	Initial content	0.88±0.03	1.08±0.05	0.59±0.04	0.88±0.05	*0.02±0.00*	*0.46±0.07*	*0.39±0.19*
	Uptake	0.85±0.08	0.97±0.06	0.66±0.25	0.78±0.06	*0.01±0.00*	*0.33±0.04*	*0.25±0.09*
	Chase	0.78±0.06	0.89±0.08	0.28±0.02	0.71±0.02	*0.01±0.00*	*0.26±0.06*	*0.30±0.10*
Δ*cobW3*	Initial content	0.89±0.03	0.94±0.05	0.62±0.05	0.77±0.01	*0.01±0.00*	*0.49±0.04*	*0.44±0.02*
	Uptake	0.83±0.11	0.91±0.02	0.58±0.14	0.71±0.02	*0.01±0.00*	*0.34±0.03*	*0.36±0.02*
	Chase	0.86±0.03	0.85±0.17	0.30±0.04	0.68±0.02	*0.01±0.00*	*0.31±0.02*	*0.41±0.04*
Δ*zupT* Δ*cobW2*	Initial content	0.76±0.04	1.02±0.11	0.65±0.11	0.92±0.04	*0.02±0.00*	*0.42±0.02*	*0.19±0.04*
	Uptake	0.87±0.07	0.95±0.03	0.54±0.07	0.83±0.02	*0.01±0.00*	*0.30±0.04*	*0.25±0.10*
	Chase	0.84±0.08	0.84±0.06	0.56±0.26	0.80±0.04	*0.01±0.00*	0.79±0.69	*0.51±0.32*
Δ*zupT* Δ*cobW3*	Initial content	0.72±0.01	1.00±0.10	0.75±0.10	0.74±0.01	*0.02±0.00*	1.23±0.55	*0.60±0.35*
	Uptake	0.73±0.03	0.93±0.13	0.64±0.02	0.70±0.01	*0.02±0.00*	0.72±0.02	*0.57±0.33*
	Chase	0.72±0.01	0.82±0.05	0.70±0.25	0.68±0.02	*0.02±0.00*	0.83±0.25	*0.63±0.32*

[a]This table shows the metal content of the metals other than zinc stemming from the ^{67}Zn pulse-chase experiments, the initial metal content, after the uptake period of 20 min and after the chase period of 40 min. Shown is the ratio compared to the metal content of strain AE104 cells grown in zinc-replete mZn medium. Bold and italics < 67%, Bold > 150%. The Mn values were close to the detection limit and not considered. The Co values from cells cultivated in lZn_lMg and lZn media just in italics. The data for strains AE104 and Δ*zupT* were published and obtained in the same experimental series as the other data (5).

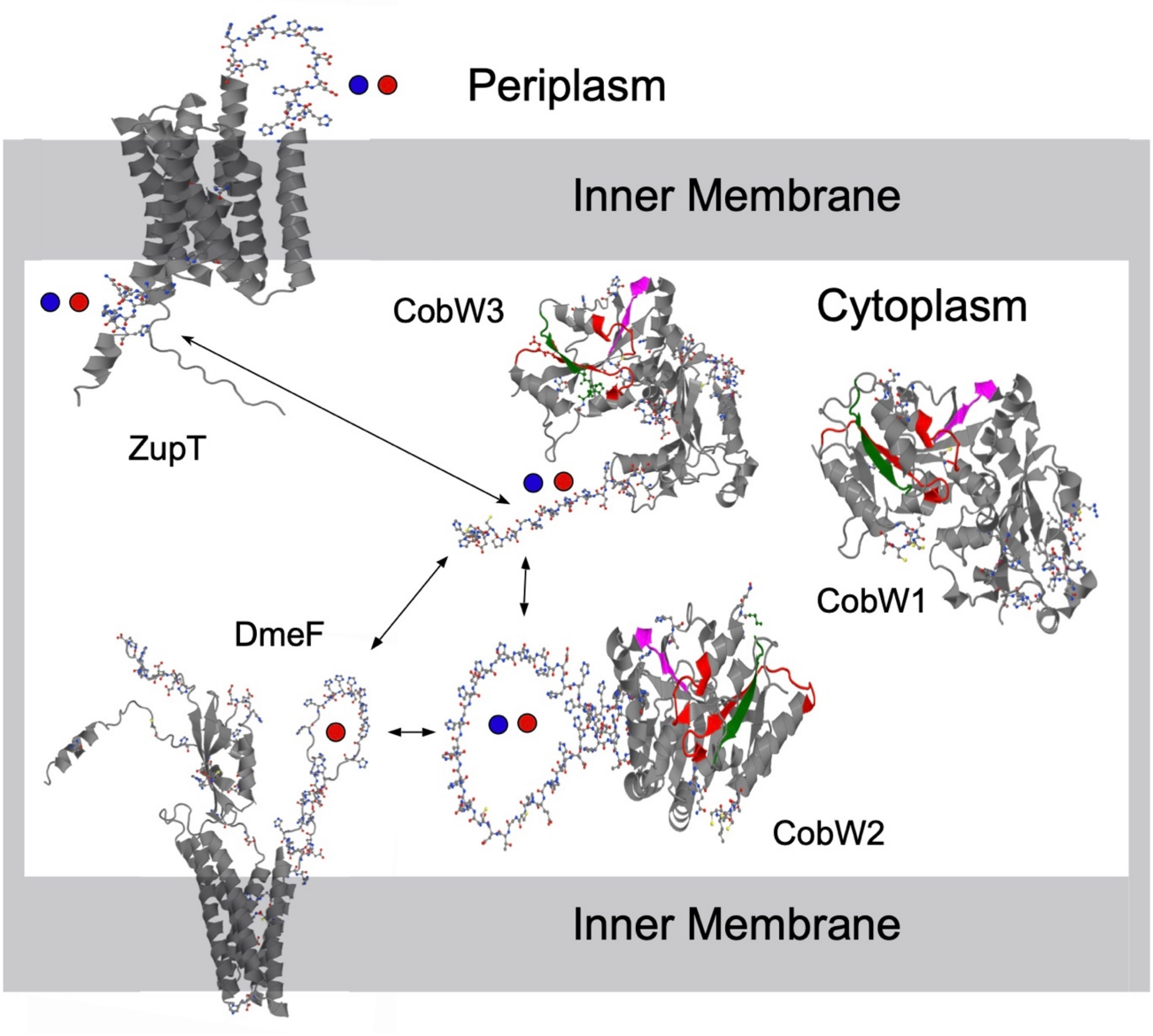

Supplementary Figure S1. The main actors: structural models of the proteins involved in interlinked zinc and cobalt homeostasis in *C. metallidurans*. AlphaFold2-generated structural models (6) of the metal ion importer ZupT, the exporter DmeF and their interacting partners CobW2 and CobW3 are shown. Although not the subject of this publication, CobW1 was added for reference. In the CobW structure predictions, the Walker regions A and B are in red, the G-binding site in purple and the switch motif in green. Below the alpha-helix opposite to the Walker domains the internal metal-binding site is shown, which is absent in CobW3. For all proteins, His-rich regions were indicated in the “ball-and-stick” representation. This highlights the large internal His-rich region in CobW2, the C-terminal one in CobW3, as well as the cytoplasmic and periplasmic His-rich regions in DmeF and ZupT. Arrows indicate the interplay of ZupT, DmeF, CobW2 and CobW3, which concerns the linked zinc and cobalt homeostasis as indicated by the blue and red filled circles, respectively. Please consider that ZupT is one out of at least 10 possible uptake systems for divalent metal cations in *C. metallidurans*. Consequently, there is still import of all kinds of metal cations in the ∆*zupT* mutants.

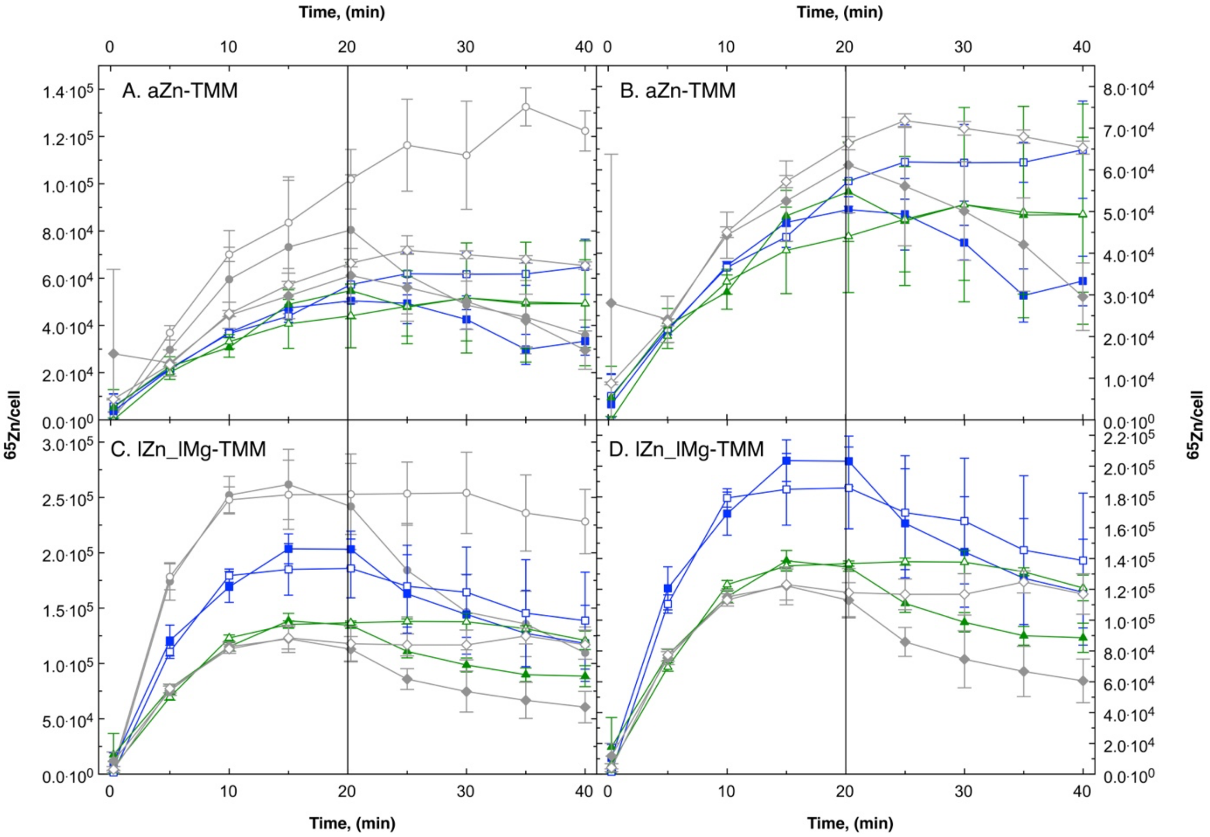

Supplementary Figure S2. Pulse-chase experiment with *C. metallidurans* strains AE104 and ∆*cobW3* mutants. Cells of strain AE104 (grey circles), ∆*zupT* (grey diamonds), ∆*cobW3* (blue squares) and ∆*zupT* ∆*cobW3* (green triangles) were cultivated in TMM containing 200 nM Zn(II) as ambient zinc medium (aZn-TMM, Panels A and B) or no added Zn(II) and 0.1 mM Mg(II) (lZn_lMg, Panels C and D) as described in Fig. 2. Pulse at t = 0 with 1 µM ^{65}Zn(II), chase at t = 20 min with 100 µM non-radioactive Zn(II) (black symbols), or not chased (open symbols). Panels B and D contain the same data as Panels A and C with the exception of the AE104 values. The data for strains AE104 and ∆*zupT* were already published (5) and obtained in the same experimental series as the other data.

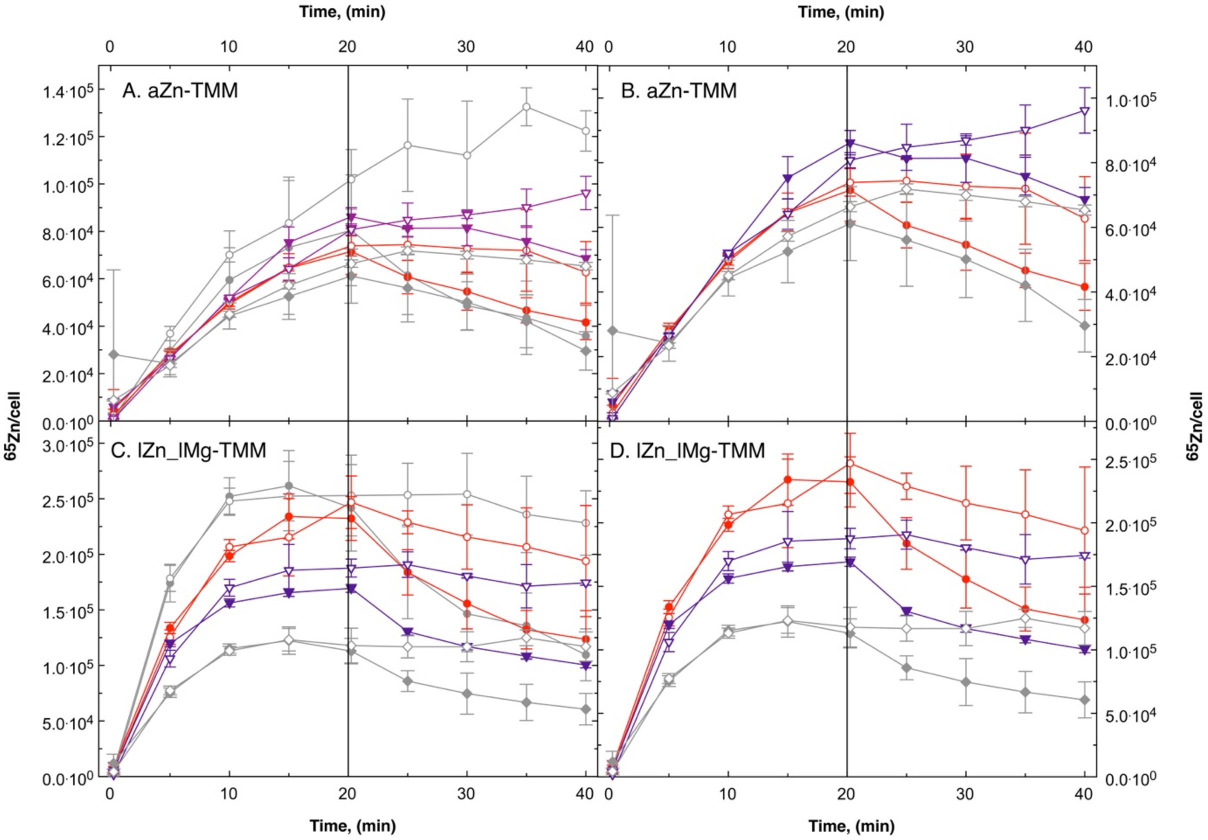

Supplementary Figure S3. Pulse-chase-experiment with *C. metallidurans* strains AE104 and ∆*cobW2* mutants. Cells of strain AE104 (grey circles), ∆*zupT* (grey diamonds), ∆*cobW2* (red circles) and ∆*zupT* ∆*cobW2* (purple inverted triangles) were cultivated in TMM containing 200 nM Zn(II) as ambient zinc medium (aZn-TMM, Panels A and B) or no added Zn(II) and 0.1 mM Mg(II) (lZn_lMg, Panels C and D) as described in Fig. 2. Pulse at t = 0 with 1 µM ^{65}Zn(II), chase at t = 20 min with 100 µM non-radioactive Zn(II) (black symbols), or not chased (open symbols). Panels B and D contain the same data as Panels A and C with the exception of the AE104 values. The data for strains AE104 and ∆*zupT* were already published (5) and obtained in the same experimental series as the other data.

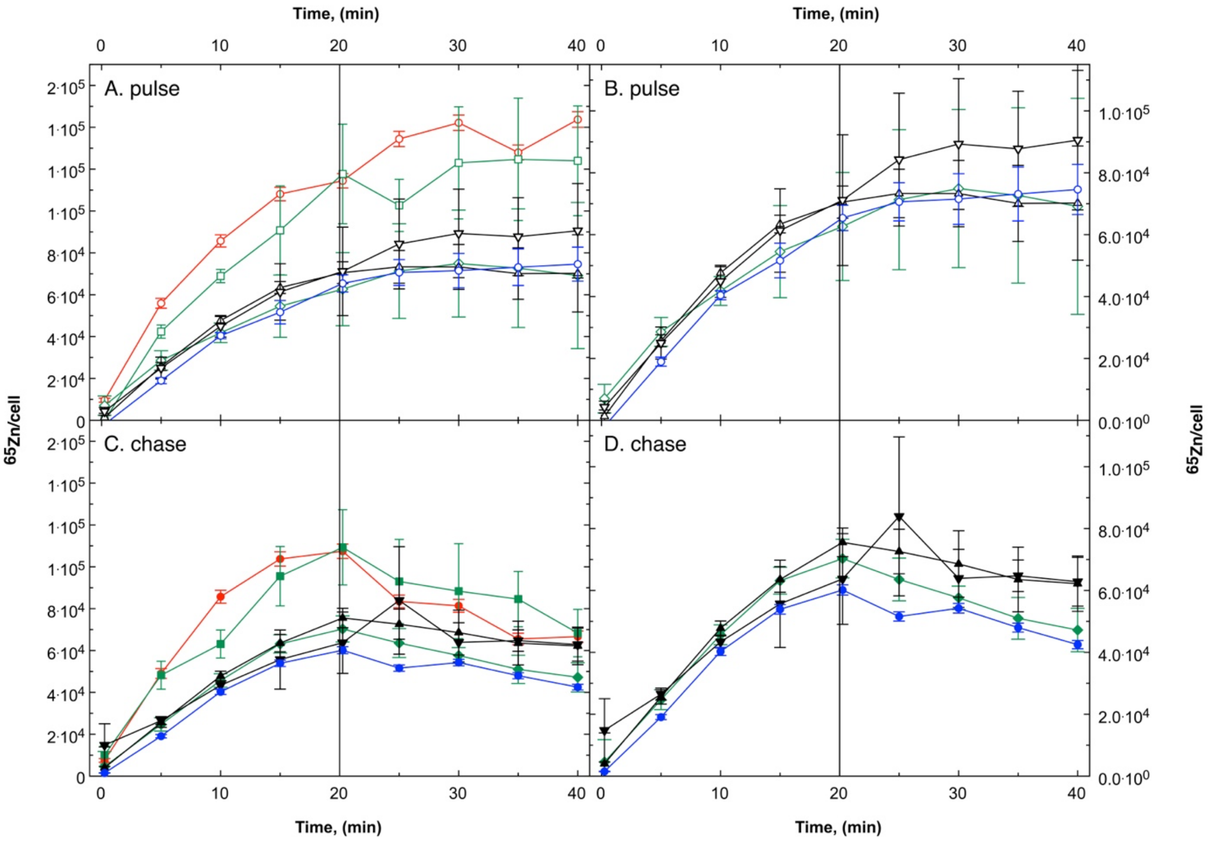

Supplementary Figure S4. Pulse-chase experiment with *C. metallidurans* strains AE104 and ∆*cobW* mutants. Cells of strain AE104 (red circles), ∆*zupT* (blue circles), ∆*cobW2* (green squares), ∆*cobW3* (green diamonds), ∆*zupT* ∆*cobW2* (black triangles) and ∆*zupT* ∆*cobW3* (black inverted triangles) were cultivated in lZn-TMM containing no added Zn(II) and 1 mM Mg(II) as described in Fig. 2. Pulse at t = 0 with 1 µM ^{65}Zn(II), Chase at t = 20 min with 100 µM non-radioactive Zn(II) (closed symbols, Panels C and D), or not chased (open symbols, panels A and B). Panel B contains the data from Panel A and Panel D those from Panel C but without the AE104 and ∆*cobW2* data. The data for strains AE104 and ∆*zupT* were already published (5) and obtained in the same experimental series as the other data.

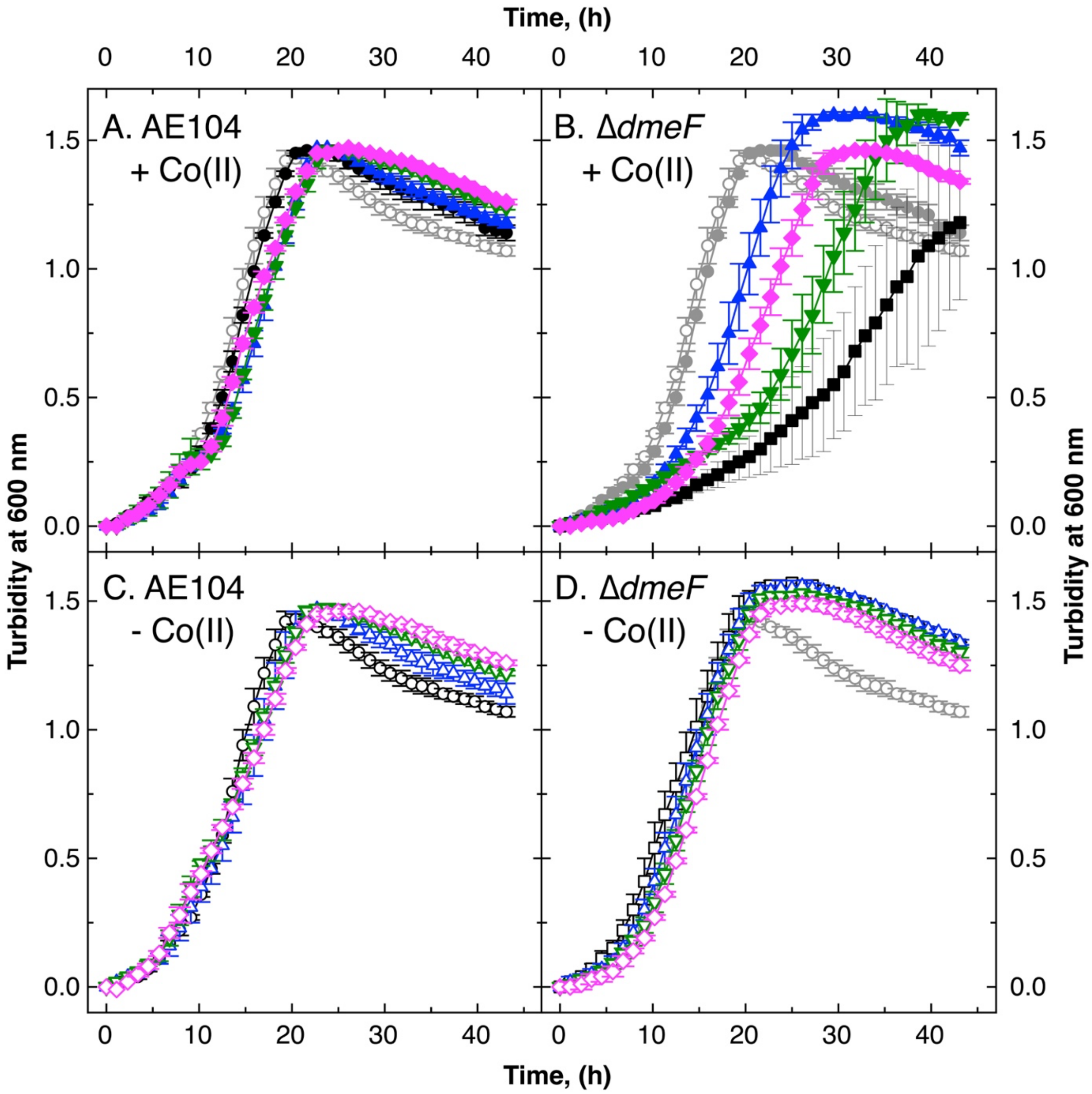

Supplementary Figure S5. CobW2 and CobW3 are responsible for growth retardation in the Δ*dmeF* mutant. Time-dependent growth is shown in standard TMM medium with 2.5 µM Co(II) in Panels A and B (closed symbols) and without added cobalt in Panels C and D (open symbols). Panels A and C show the parent strain AE104 (circles) and Panels B and D the Δ*dmeF* strain (black squares) with their respective Δ*cobW3* (blue triangles), Δ*cobW2::dis* (green inverted triangles) and Δ*cobW3* Δ*cobW2::dis* (magenta diamonds) mutants. The AE104 data are shown in Panels B to D for reference in grey. Three repeats, deviations shown.

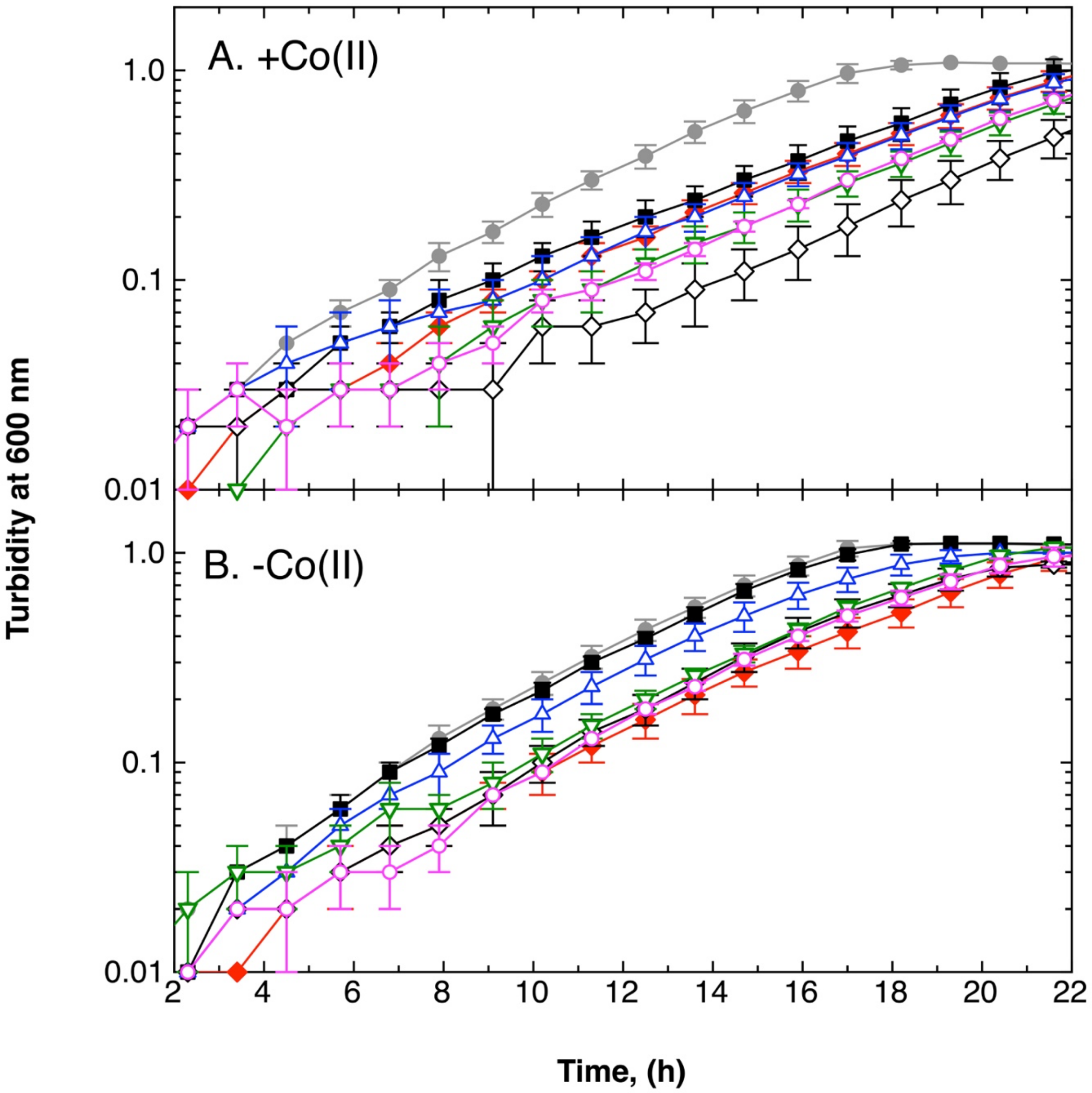

Supplementary Figure S6. Effect of Co(II) on a double deletion strain ∆*zupT* ∆*dmeF*. Time-dependent growth of strains ∆*zupT* (red filled diamonds), ∆*dmeF* (black filled squares), ∆*dmeF* ∆*zupT* (open diamonds), ∆*dmeF* ∆*zupT* ∆*cobW2* (green open inverted triangles), ∆*dmeF* ∆*zupT* ∆*cobW3* (blue open triangles) and ∆*dmeF* ∆*zupT* ∆*cobW2* ∆*cobW3* (magenta open circles) in standard TMM medium without (Panel B) or with (Panel A) 1 µM Co(II). Growth of the parent AE104 (closed circles) and is shown in grey for references. Three repeats, deviations indicated. This is Suppl. Fig. S5 but in a half-logarithmical plot for the time period between 2 h and 22 h.

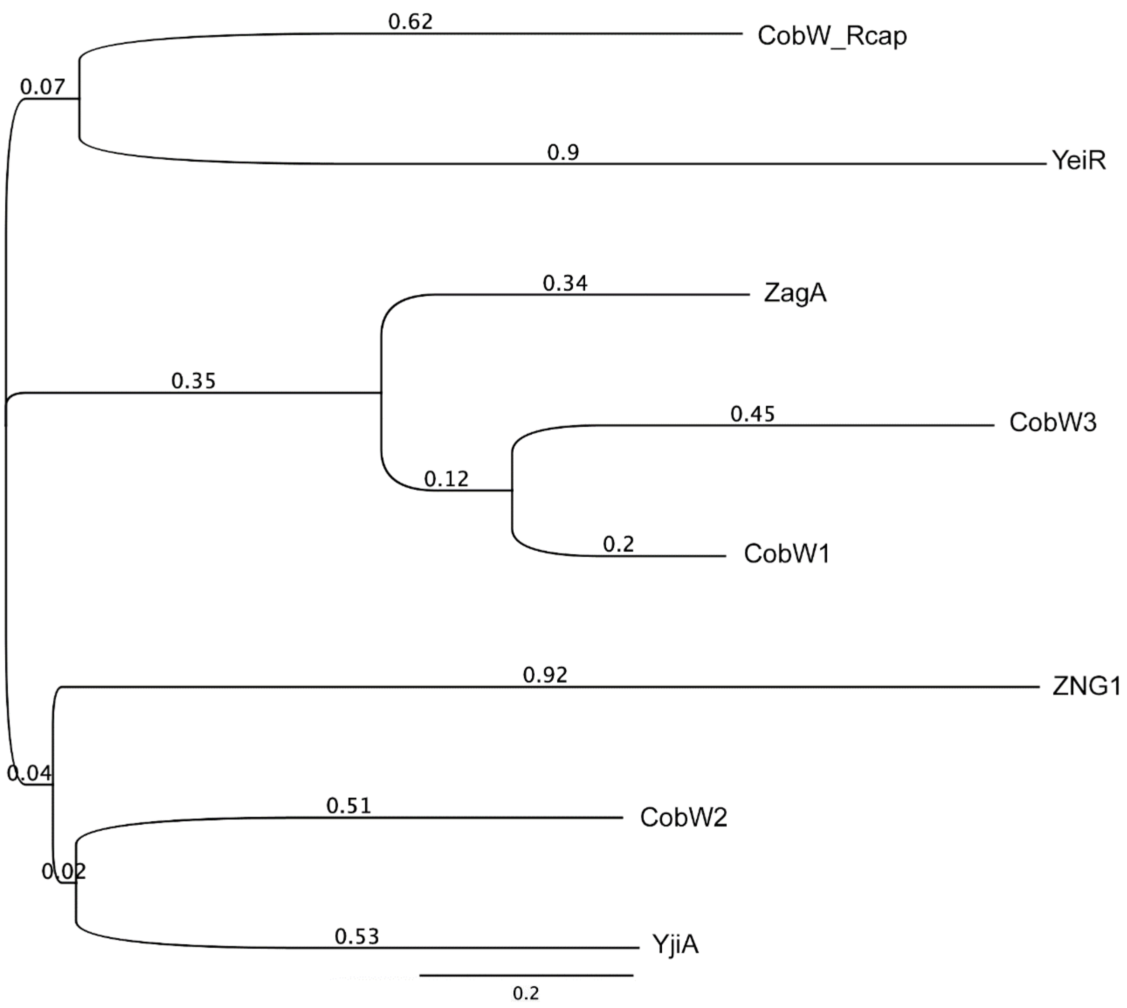


```
                   1        10        20        30        40        50        60
                   |        |         |         |         |         |         |
Rmet_0125 CobW3    MAV---------------------------------------------------------
Rmet_0127 CobW2    MSKL--------------------------------------------------------
Rmet_1098 CobW1    MLPA--------------------------------------------------------
           CobW    MSDLT-------------------------------------------------------
           YeiR    MTR---------------------------------------------------------
           YjiA    MNP---------------------------------------------------------
    ZagA = YciC    MKK---------------------------------------------------------
           ZNG1    MSALRNIKYNEEEDGELPSLVTGEEDNLQEILENVSYDGGNIVSDAKVERVNKQVESVSK

Rmet_0125 CobW3    --------R-LPVTVLSGFTGAGKTTVLRHL-EAQA-GARVAVVDSH-------------
Rmet_0127 CobW2    ----------IPVTILTGFLGSGKTTLLKRILNEQH-GMKIAVIENEFGEEN-IDNEILV
Rmet_1098 CobW1    ---------KLPVTVLSGFLGAGKTTLLNHILHNRE-GRRVAVIVNDMSDVN-IDAGLVR
           CobW    ---------KIPVTVITGFLGAGKTTLIRHLMANPE-GRKLAVLVNEFGTVG-VDGEILR
           YeiR    ------------TNLITGFLGSGKTTSILHLLAHKDPNEKWAVLVNEFGEVG-IDGALLA
           YjiA    ----------IAVTLLTGFLGAGKTTLLRHILNEQH-GYKIAVIENEFGEVS-VDDQLIG
    ZagA = YciC    ----------IPVTVLSGYLGAGKTTLLNSILQNRE-GLKIAVIVNDMSEVN-IDAGLVK
           ZNG1    DMTDSQKRKRVPVSIITGYLGSGKSTLLEKIALKGA-DKKIAVILNEFGDSSEIEKAMTI
                                     Walker A                Switch
Rmet_0125 CobW3    --------------------------GDLLAEVERL-AQTGQY----DHVVIEAGATDEP
Rmet_0127 CobW2    QDG------REQIVQMSNGCICCTIRGDLVQALSDLVTQRDEGKIAFDRVVIETTGVANP
Rmet_1098 CobW1    DGGANLSRTEETLVEMSNGCICCTLREDLLLEVERL-AREGRF----DQLVIESTGISEP
           CobW    QCA-DENCPDENIVELANGCICCTVADEFIPTIEALMARPVRP----DHILIETSGLALP
           YeiR    DSG-------ALLKEIPGGCMCCVNGLPMQVGLNTLLRQGKP-----DRLLIEPTGLGHP
           YjiA    DRA-------TQIKTLTNGCICCSRSNELEDALLDLLDNLDKGNIQFDRLVIECTGMADP
    ZagA = YciC    QEG-GLSRTDEKLVEMSNGCICCTLREDLLIEVEKL-AKDGRF----DYIVIESTGISEP
           ZNG1    KNG---SSSYQEWLDLGNGCLCCSLKNIGVKAIEDMVERSPGKI---DYILLETSGIADP
                           Internal metal-binding site        Walker B
```

```
Rmet_0125 CobW3   LGLAEGFAF-EDEHGE-TTSVGHLDTLVTVIDASRFLQDYHDADFLSARALAAHD---DD
Rmet_0127 CobW2   GPVAQTFFM-DEE----IASRYLLDAVITLVDAKHANLQLDKQEE----AQR--------
Rmet_1098 CobW1   LPVAETFTF-EGEDGRSLNEVAHLDTMVTVVDAFNFLRDYGSRDSLQSRGESLGE---ED
           CobW   KPLLKAFDW-PA-----IRSKITVDGVIAVADAEAVAAGRFAPDVAAVDAQRQADDIIDH
           YeiR   KQILDLLTA-PV-----YEPWIDLRATLCILDPRLLLDEKSASN------EN--------
           YjiA   GPIIQTFFS-HEV----LCQRYLLDGVIALVDAVHADEQMNQFT-----IAQ--------
    ZagA = YciC   IPVAQTFSYIDEEMGIDLTKFCQLDTMVTVVDANRFWHDYQSGESLLDRKEALGE---KD
           ZNG1   APIAKMFWQDEGL-----NSSVYIDGIITVLDCEHILKCLDDVSADAHWHGDKV-----G

Rmet_0125 CobW3   DRTVVDVLIDQVEVCDVLVVNKIDLV-DAGQ-LGRLHVMLHALNPRA-DIVDASHGQVPA
Rmet_0127 CobW2   ----------QVGFADAIFITKADLVS-EAE-VADLRHRLLHMNPRA-PINAAHFGEAPI
Rmet_1098 CobW1   ARTVVDLLIDQVEFCDVLVLNKVDLI-ADAD-RERLLAILRALNPRA-RIEIAEFGRVPL
           CobW   ETPLSEVFEDQIACADIVLLSKADLA-GAEG-LATARALIEAELPRKLPILPLTEGVIDP
           YeiR   -------FRDQLAAADIIVANKSDRT--TPE-SEQALQRWWQQNGGDRQLIHSEHGKVD-
           YjiA   ---------SQVGYADRILLTKTDV---AGE-AEKLHERLARINARA-PVYTVTHGDIDL
    ZagA = YciC   EREIADLLIDQIEFCDVLILNKCDLVS-EQE-LEQLENVLRKLQPRA-RFIRSVKGNVKP
           ZNG1   LGGNLTIAHFQLAMADRIIMNKFDNIEHSPEAVENLKKRVREINSIA-PMYFTKYGETSI
                                  G-binding
Rmet_0125 CobW3   ARVLDTGRFDIDATPNAAGWQAA------LQGDTVPDAAGVSTLVYRRRRPFHPQRFADL
Rmet_0127 CobW2   DLIFDLRGFNLNEKLEIDPDFLRADEHDHEHDHEHGEHCGTDCGHDHGHEHGHDHGHGHA
Rmet_1098 CobW1   ERVLGTGLFDFEEASKAPGWLQEM----RGQHVPETEEYGIRSFVYGARRPFHPQRFHAF
           CobW   KVILGLGAAAEDDLAARPS---------HHDDHDDHEHDDFDTVVIELPEIADPAALVAA
           YeiR   GHLLDLPRRNLAELPASAA---------HSHQHVVKKGLAALSLPEHQRWRRSLNSGQGY
           YjiA   GLLFNTNGFMLEENVVSTK---------PRFHFIADKQNDISSIVVELDYPVDISEVSRV
    ZagA = YciC   QEILHTGLFNFEEASGSAGWIQELTA-GHAEHTPETEEYGISSFVYKRRLPFHSTRFYRW
           ZNG1   DNLLDIHAYDSIRISDILDNKVEE----GTIHDHRMSTVTLTFRPLKDEEEYNEKFLKQF
                                                 His-stretch in CobW2
Rmet_0125 CobW3   IHTEWMREHGDV------LRSKGLFWLASRMDIAGDWSQAGGVCRPGAAGAWWAAIDAEE
Rmet_0127 CobW2   HDHHHHHHHTDRIASFVFRSDKPFHYGKLEEFLSGILSVYGEKLLRYKGVLYMEGVDRKV
Rmet_1098 CobW1   VAAEWPGV----------VRSKGFFWLASHPTLAGTWSQAGAVARHGPAGYWWAAVPPER
           CobW   IERLAREQN--------ILRVKGHIAVAGKP-MRLLVQAVGERVRHQYDRPWGTEAR---
           YeiR   QACGWIFDADTV---FDTIGILEWARLAPVERVKGVLRIPEGLVRINRQGDDLHIETQNV
           YjiA   MENLLLESADKL------LRYKGMLWIDGEPN-RLLFQGVQRLYSADWDRPWGDEKPH--
    ZagA = YciC   LDQMPKNV----------VRAKGIVWCASHNNLALLMSQAGPSVTIEPVSYWVAALPKLE
           ZNG1   LQPLLWKNFGAMTVLKGDLQHKERGWEVQRTKGLVLIEGEKLTTRVIQGVRDTYDVFPGE

Rmet_0125 CobW3   WPPEGPARAAIEADLIEDGLPAEYGDRRQELVLIGLDL-DAHALEAQLDACLLTDDEMAA
Rmet_0127 CobW2   VFQGVHQLM-----GSDIGAKWEDEQPGTKMVFIGVDL-PRDAILKGLAACLA-------
Rmet_1098 CobW1   WPDDPESVA-----LIRAKWDDSVGDARQEIVLIGVDM-DEFELRRCLDACLLTDDEMAQ
           CobW   -----RS---------------------ALVVIAEHHDVDEAAIRAVLLGGVAA------
           YeiR   APPD--S----------------------RIELISSSEADWNALQSALLKLRLATTA---
           YjiA   ------S----------------------TMVFIGIQL-PEEEIRAAFAGLRK-------
    ZagA = YciC   QEQVKQQEP-----EILEEWDPEFGDRLTQLVFIGTDL-DEETITKELDQCLLTEYEFDS
           ZNG1   YNGSEKE----------C-----------KIVLIGKYL-EKDSIKKLLDKTLK-------

Rmet_0125 CobW3   GPEAWAAYPDPFPDWGNAFDDDHDHDHGHDHDHGDGPCDCGHAH
Rmet_0127 CobW2   --------------------------------------------
Rmet_1098 CobW1   GPDVWTTWHNPFPDWP----------------------------
           CobW   --------------------------------------------
           YeiR   --------------------------------------------
           YjiA   --------------------------------------------
    ZagA = YciC   D---WSLFEDPFKWKLN----------Q----------------
           ZNG1   --------------------------------------------
                                 C-terminal His-stretch in CobW3
```

Supplementary Figure S7. Multiple alignment of the CobWs with related proteins. The top shows the Tree representation of the relationship of the three CobWs of *C. metallidurans* with CobW_Rcap from *Rhodobacter capsulatus*, ZagA from *Bacillus subtilis*, ZNG1 from *Saccharomyces cerevisiae* and other proteins. Walker A-, B-, switch-, G-binding-, internal metal binding and His-rich stretches are indicated. The alignment is an updated version of an alignment published previously (3).

Literature

1. Mergeay M, Nies D, Schlegel HG, Gerits J, Charles P, van Gijsegem F. 1985. *Alcaligenes eutrophus* CH34 is a facultative chemolithotroph with plasmid-bound resistance to heavy metals. J Bacteriol 162:328-334.
2. Kirsten A, Herzberg M, Voigt A, Seravalli J, Grass G, Scherer J, Nies DH. 2011. Contributions of five secondary metal uptake systems to metal homeostasis of *Cupriavidus metallidurans* CH34. J Bacteriol 193:4652-4663.
3. Bütof L, Große C, Lilie H, Herzberg M, Nies DH. 2019. Interplay between the Zur regulon components and metal resistance in *Cupriavidus metallidurans*. J Bacteriol 201:e00192-19.
4. Scherer J, Nies DH. 2009. CzcP is a novel efflux system contributing to transition metal resistance in *Cupriavidus metallidurans* CH34. Mol Microbiol 73:601-621.
5. Nies DH, Schleuder G, Galea D, Herzberg M. 2024. A flow equilibrium of zinc in cells of *Cupriavidus metallidurans*. J Bacteriol 5:10.1128/jb.00080-24.
6. Varadi M, Anyango S, Deshpande M, Nair S, Natassia C, Yordanova G, Yuan D, Stroe O, Wood G, Laydon A, Žídek A, Green T, Tunyasuvunakool K, Petersen S, Jumper J, Clancy E, Green R, Vora A, Lutfi M, Figurnov M, Cowie A, Hobbs N, Kohli P, Kleywegt G, Birney E, Hassabis D, Velankar S. 2021. AlphaFold Protein Structure Database: massively expanding the structural coverage of protein-sequence space with high-accuracy models. Nucleic Acids Research 50:D439-D444.
